# Climate hazards expose distinct compliance vulnerabilities in US drinking water systems

**DOI:** 10.21203/rs.3.rs-9217282/v1

**Published:** 2026-04-15

**Authors:** Chris C. Lim

**Affiliations:** Department of Community, Environment and Policy, Mel & Enid Zuckerman College of Public Health, University of Arizona, Tucson, AZ, USA

## Abstract

Climate extremes threaten drinking water systems. Whether climate hazards compromise drinking water regulatory compliance, and through which pathways, remains uncharacterised at national scale. We linked extreme precipitation, heat intensity, and wildfire smoke PM_2.5_ to monthly Safe Drinking Water Act violation risk across twelve contaminant categories in 56,351 community water systems (2006–2020). Extreme precipitation produced the broadest disruption, increasing turbidity, coliform, disinfection byproduct and treatment-technique violations by 8–13%, concentrated in surface-water and smaller systems. Heat showed a narrower, microbial-specific signal: *Escherichia coli* (*E. coli*) violation risk increased ~2.6% per °C, though the pathway could not be mechanistically isolated. Wildfire smoke associations were fragile: pooled estimates attenuated under strict seasonal controls, and only an exploratory transport-distance probe remained positive. These findings reveal a graded evidence hierarchy, precipitation robust, heat selective, smoke provisional, arguing for climate-responsive monitoring triggers matched to hazard-specific evidence strength, including post-precipitation follow-up for smaller systems.

## Introduction

More than 300 million Americans receive drinking water from over 148,000 public water systems (PWSs) regulated under the Safe Drinking Water Act^1^. These systems face compounding climate pressures that may compromise their capacity to maintain regulatory compliance. Anthropogenic climate change has already doubled the area burned by wildfire across the western United States^2^, extreme heat events have grown more frequent and prolonged^3^, and extreme precipitation has intensified across the continental US^4^. Each can degrade source water quality through distinct mechanisms^5–8^. Yet the existing evidence base is fragmented. Most studies examine a single hazard in a single region^9–12^. Critically, nearly all measure source water quality rather than regulatory compliance at the point of delivery. Source water monitoring isolates contamination, but compliance violations capture the full system response: whether treatment, infrastructure, and oversight absorbed the stress or failed under it. Whether source water effects translate into compliance failures through the same or different pathways remains uncharacterised at national scale, a gap recently identified as a research priority^13^.

Safe Drinking Water Act (SDWA) violations offer a particularly informative outcome for addressing this question. Unlike ambient water quality monitoring, violations integrate treatment capacity, infrastructure condition, and source water quality into a single policy-relevant indicator. SDWA violations span twelve categories, from microbial indicators (*E. coli*, total coliform, turbidity) to chemical contaminants (arsenic, nitrate, disinfection byproducts) to operational categories (treatment technique, monitoring and reporting). Because some of these endpoints overlap by rule structure and are monitored on different cadences, we treat this twelve-category analysis as a broad screening framework rather than as twelve independent mechanistic tests.

**Figure 1 |:**
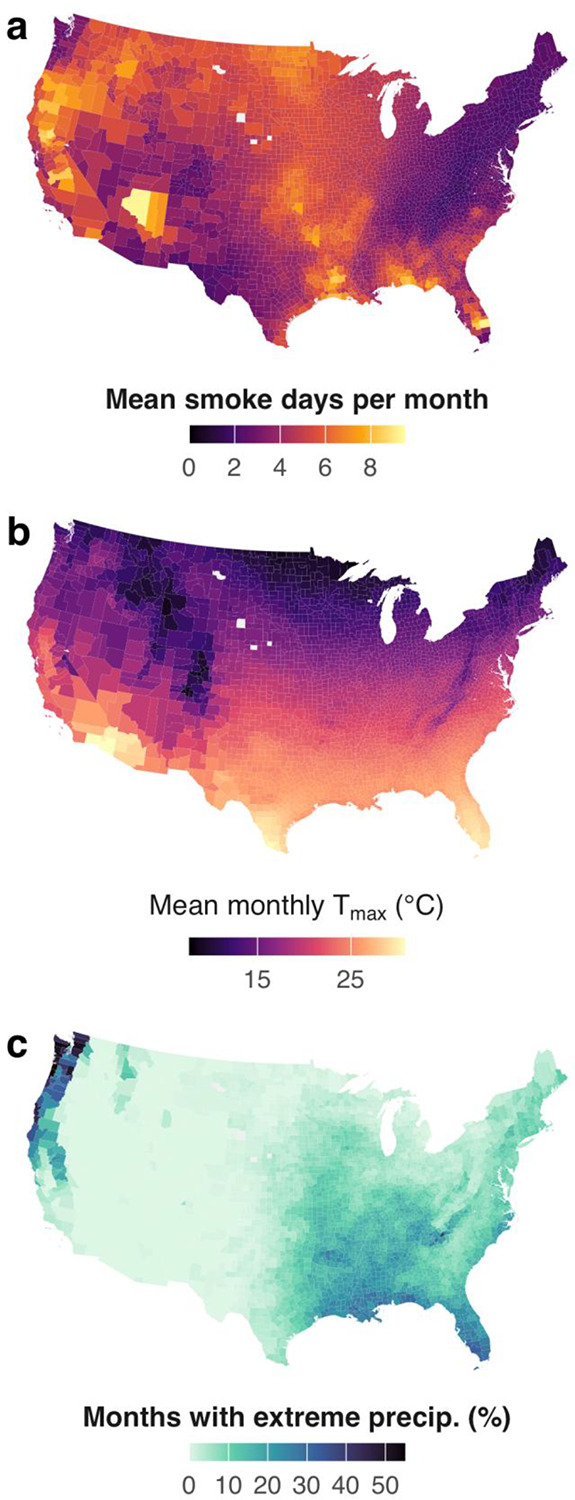
Spatial distribution of climate hazard exposures. **a**, Wildfire smoke exposure (mean smoke days per month, 2006–2020). **b**, Heat exposure (mean monthly maximum temperature, °C). **c**, Extreme precipitation frequency (percentage of months exceeding county-specific 90th percentile).

We apply this screening framework to 56,351 contiguous-US community water systems from 2006 to 2020 (of ~97,000 CWSs in SDWIS, ~33,000 are inactive historical entries with no operating data, and ~7,000 lack CONUS location or county linkage; see Methods), linking PRISM temperature and precipitation records, daily wildfire smoke PM_2.5_ estimates^14^, and Monitoring Trends in Burn Severity (MTBS) fire data to monthly SDWA violation records across three climate hazards (Figure 1): extreme precipitation, heat, and wildfire smoke, with compound co-occurrence as a secondary diagnostic. Each pathway is screened against all twelve violation categories and stratified by water source type, with the most comparable monthly microbial outcomes carrying the most interpretive weight. The central question is not simply whether climate hazards affect drinking water in aggregate, but whether they generate a graded hierarchy of compliance-risk patterns, monitoring windows, and operational implications. We validate the violation-based findings against measured water quality concentrations from the EPA/USGS Water Quality Portal, with validation strength varying across hazards.

## Results

### Precipitation drives the broadest compliance disruption

**Figure 2 |:**
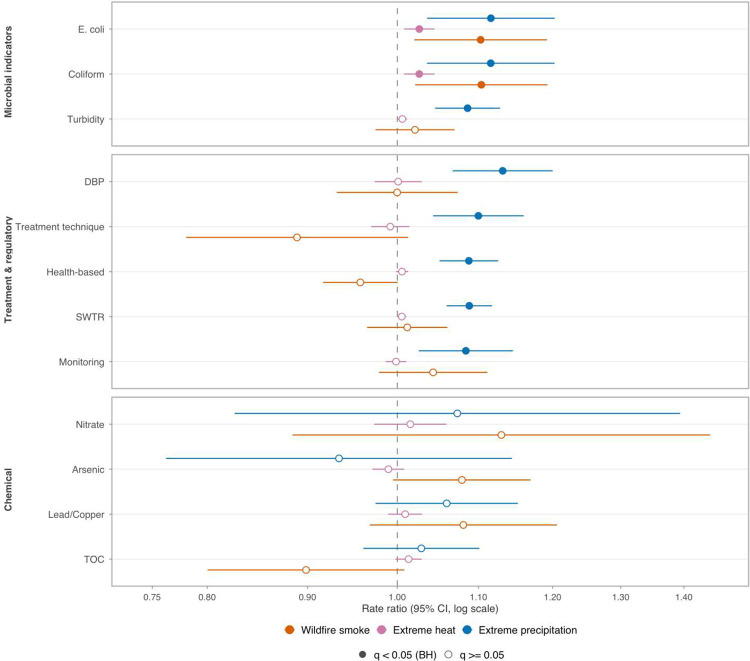
Climate hazard screening across drinking water violation categories. Forest plot showing rate ratios (95% confidence intervals) for smoke exposure (≥10 days), heat intensity (per °C), and extreme precipitation (>P90) across twelve violation categories. This panel is intended as a broad specificity screen rather than a set of independent mechanistic tests: *E. coli* and coliform overlap under the Total Coliform Rule, health-based violations aggregate multiple rules, and some categories are monitored on slower cadences than the monthly microbial outcomes. Microbial indicators (*E. coli*, coliform, turbidity) have the clearest acute event pathways; treatment and regulatory violations (DBP [disinfection byproducts], treatment technique, health-based, SWTR [Surface Water Treatment Rule], monitoring) are more operationally mediated; chemical contaminants (nitrate, arsenic, lead/copper, TOC) have less plausible acute-event pathways. Filled circles indicate q < 0.05 (Benjamini-Hochberg corrected); hollow circles indicate q ≥ 0.05. One CI (arsenic × precipitation) extends beyond the plotted range. All models use PWS and year-month fixed effects with state-clustered standard errors. 12 of 36 screened associations survive FDR correction at q < 0.05.

The analytic sample comprised 56,351 community water systems observed over 9.0 million system-months (Table 1). Across the twelve-category screening analysis (Figure 2), the three hazards showed a graded hierarchy: precipitation broad and robust, heat narrower but reproducible, smoke selective and identification-sensitive (Table 2). Because some outcomes overlap by construction and are monitored on different cadences, we pre-designate three microbial endpoints (turbidity, total coliform, *E. coli*) as primary and treat the remaining nine categories as a secondary screening panel.

**Figure 3 |:**
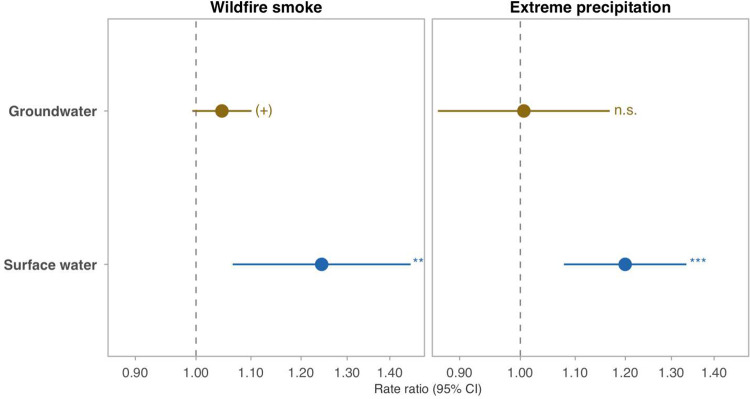
Source-type stratification is clearer for precipitation than for smoke. Two hazard-specific dot-whisker panels compare surface-water and groundwater estimates directly. The precipitation panel (PWS-level total violations, Design 3) shows a clearer surface-water concentration. The smoke panel is a secondary county-level turbidity diagnostic rather than the primary smoke result in the manuscript; in that simpler stratification the surface-water point estimate is larger, but alternative smoke source-architecture analyses do not confirm a stable surface-water interaction (Supplementary Table 5). We therefore interpret the smoke source-type pattern as suggestive rather than as firm evidence of a surface-water deposition mechanism. Heat is omitted because the county-level DLNM (Design 2) aggregates across source types within each county. Significance labels: ***P < 0.001, **P < 0.01, (+) *P* < 0.10, n.s. *P* >= 0.10.

Extreme precipitation elevated violations across microbial, operational, and regulatory categories (Figure 2): turbidity and coliform increased 8–13%, with parallel increases in disinfection byproduct, treatment-technique, and health-based violations (turbidity RR = 1.086; *P* < 0.001; ~9 additional violations per 10,000 system-months above the baseline rate). The effect concentrated in surface water systems (RR = 1.200; *P* < 0.001; Figure 3) and was robust to every level of seasonal control (Extended Data Table 4), but population weighting attenuated the turbidity estimate to non-significance (RR = 1.053; *P* = 0.20), indicating that the signal is concentrated in smaller systems.

Daily within-site Water Quality Portal (WQP) models confirmed acute responses: same-day *E. coli* rose 68.2% (*P* < 0.001) and turbidity rose 52.8% over a 0–7 d window (*P* < 0.001), with steeper slopes at flowing than standing sites (Supplementary Tables 8–9).

### Heat: secondary monthly heat-intensity signal

Heat showed a selective microbial signature. *E. coli* violations increased 1.026 per °C (*P* = 0.004; ~0.23 additional violations per 10,000 system-months per °C), but the accompanying validation pattern was mixed rather than mechanistically resolved. Under the strictest seasonal specification, the estimate remained 1.031 per °C (*P* < 0.001). By contrast, threshold-defined hot-month indicators were null or weakly negative in the same PWS fixed-effects *E. coli* model (P90 RR = 0.911, *P* = 0.08; P95 RR = 0.933, *P* = 0.22; Extended Data Table 6), reinforcing that the main heat result is a monthly heat-intensity sensitivity rather than a discrete extreme-month effect. In post hoc source-architecture stratification, the heat-*E. coli* association was present in groundwater systems (RR = 1.029; *P* = 0.004) but null in surface systems (RR = 0.999; *P* = 0.96), with a significant surface-versus-groundwater interaction (RR = 0.973; *P* < 0.001; Supplementary Table 3).

Daily within-site models showed short-window heat increases in *E. coli* (41.1%; *P* < 0.001) and turbidity (19.9%; *P* = 0.005) over 0–14 d windows (Supplementary Table 9). A county-level distributed lag non-linear model (DLNM) for composite violations yielded a smaller cumulative RR of 1.037 (95% CI: 0.960–1.120), reflecting signal dilution (Supplementary Figure 3); the outcome-specific *E. coli* DLNM peaked at lag 0 and was significant only through lags 0–2 (Extended Data Table 6). These results identify a reproducible heat-related microbial signature, but the current probes do not establish whether the pathway is treatment stress, distribution-system conditions, or reporting structure.

### Wildfire smoke: exploratory transport probe

In the 12-outcome screen, pooled smoke associations were limited to the two microbial categories (*E. coli* and coliform), but only the *E. coli* association carried into the distance-based identification analyses below. Wildfire smoke was associated with elevated *Escherichia coli* violations at the PWS level under the primary specification (RR = 1.098; *P* = 0.02), but this pooled association was sensitive to seasonal controls: it was attenuated to null under county × month-of-year fixed effects alone (RR = 1.116; *P* = 0.11; 55% attenuation). Because fire season in the western US peaks during summer months (when water demand, workforce turnover, baseline temperature, and source water chemistry also change), seasonal confounding is a plausible alternative explanation for the pooled result.

To separate atmospheric smoke from these co-occurring stressors, we used geographic variation in the distance between smoke exposure and the nearest active wildfire (Figure 5). *E. coli* violation risk was elevated in the 200–500 km bin (RR = 1.261; *P* < 0.001) and remained elevated at 500–1,000 km, but was null among counties within 100 km (RR = 1.020; *P* = 0.74), where local fire effects (ash runoff, infrastructure disruption) would be strongest, though monitoring disruption during local fire emergencies could also contribute to the null at short distances. This transport-distance pattern is more compatible with a transported-smoke signal than with a uniform seasonal confounder, although the distance bins were not pre-registered, the zone boundaries are researcher-chosen, and the pattern does not by itself rule out all alternative seasonal explanations. We therefore treat the transport gradient as a consistency check rather than a quasi-experimental identification.

An external WQP sampling-intensity check showed only modest local monitoring reduction within 100 km, with no comparable decline in the transport zone (Supplementary Table 2), weakening a simple monitoring-collapse explanation for the transported-smoke estimates.

Restricting to the 200–1,000 km transport zone (611,785 system-months, 90.4% of the full *E. coli* sample), the estimate attenuated under county × month-of-year effects alone (RR = 1.116; *P* = 0.11) but remained positive with weather controls (RR = 1.188; *P* = 0.004). Because adding covariates can induce suppression in high-dimensional fixed-effects settings, we treat this as suggestive rather than definitive. Source-type stratification was mixed, with no significant surface-versus-groundwater interaction (Supplementary Table 3).

### Outcome specificity and multiple testing

Monitoring violations showed no response to smoke (*P* = 0.29) or heat (*P* = 0.87) but were elevated for precipitation, consistent with the Surface Water Treatment Rule’s mandate for additional sampling; the null for smoke and heat supports specificity but could also reflect event-driven disruption (see Discussion). Chemical contaminants were generally null, though arsenic showed a suggestive smoke association (q = 0.146; Extended Data Table 1). Applying the Benjamini–Hochberg procedure across all 36 screened combinations, 12 survive at q < 0.05, predominantly precipitation effects (8 of 12); the effective number of independent precipitation tests is lower than 12 given structural overlap among turbidity, coliform, treatment technique, and SWTR outcomes. Smoke *E. coli*/coliform were marginal (q = 0.059), while heat *E. coli*/coliform survived (q = 0.013). Compound-event diagnostics found no superadditive interactions (Extended Data Table 3).

### Regional heterogeneity and demographic gradients

Regional stratification across nine Census divisions revealed geographic heterogeneity in hazard effects (Extended Data Figure 2a–b). Precipitation disruption was the most spatially uniform, while heat effects were concentrated in southern divisions and smoke effects were strongest in the Pacific and Mountain divisions. Exploratory smoke × minority interactions are reported in Extended Data Figure 2c–d and Supplementary Table 1, but several Division-level estimates (including the Mid Atlantic minority and E South Central poverty interactions) rely on only 3–4 state clusters, making cluster-robust inference unreliable regardless of nominal significance. We treat this heterogeneity block as hypothesis-generating rather than central to the main inference.

### Hazard timing suggests different monitoring windows

**Figure 4 |:**
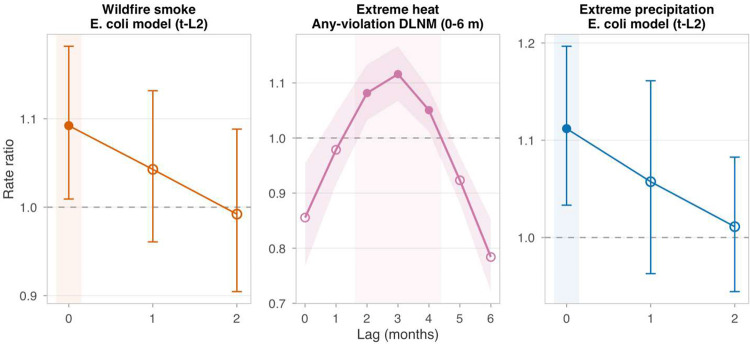
Hazard timing suggests different monitoring windows. **a**, Lag-specific relative risks for any violation at the 95th percentile of county-specific temperature from the heat DLNM, shown across 0–6 months because Design 2 uses a county-level distributed lag non-linear model. **b**, Pre-specified wildfire smoke *E. coli* lag model including only contemporaneous month, lag 1, and lag 2 (*t*, *t* − 1, *t* − 2), showing a contemporaneous-month association with no carryover. **c**, Corresponding pre-specified extreme precipitation *E. coli* lag model over the same 0–2 month window. The panels are therefore intentionally not symmetric: heat is shown as a full DLNM lag surface, whereas smoke and precipitation are shown as parsimonious outcome-specific lag models. Shaded bands mark the empirically supported monitoring window for each hazard; filled points indicate 95% confidence intervals excluding 1.

**Figure 5 |:**
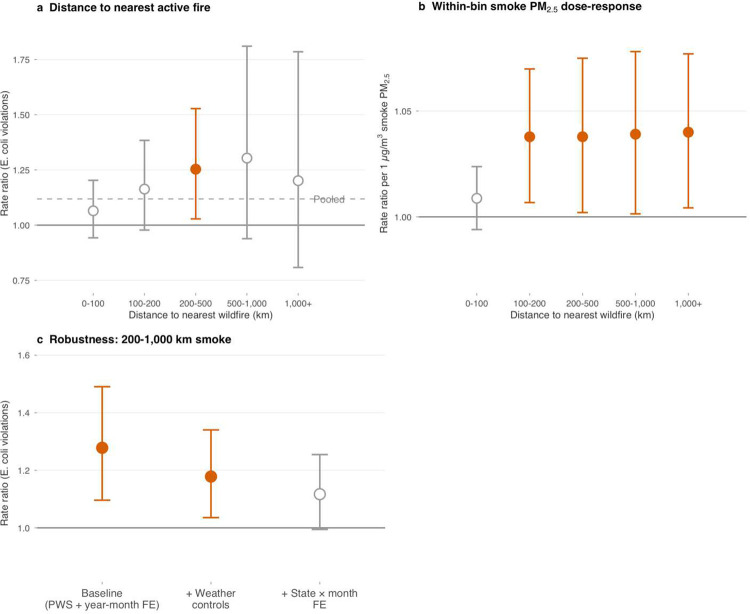
Wildfire smoke transport probe. **a**, Distance to nearest active fire gradient: rate ratios (95% confidence intervals) for binary smoke exposure (≥10 days) on *E. coli* violations, stratified by distance from county centroid to nearest MTBS burn-boundary centroid. The effect is elevated at transport distances of 200–1,000 km and null within 100 km, inconsistent with a simple local-fire or uniform seasonal explanation, but the smoke evidence overall remains more fragile than the precipitation and heat results. Dashed line shows the pooled estimate across all distances. **b**, Within-bin smoke PM_2.5_ dose–response at each distance, showing consistent per-μg/m^3^ effects at transport distances (>100 km) under baseline controls (PWS + year-month fixed effects). **c**, Robustness of the Goldilocks zone (200–1,000 km) binary smoke indicator across three specifications: baseline (PWS + year-month FE), county × month-of-year FE with weather controls, and state × month FE. Points show rate ratios; horizontal bars show 95% confidence intervals.

Hazard timing differed across pathways (Figure 4). Smoke and precipitation *E. coli* effects were concentrated in the exposure month with no carryover (1-month and 2-month lag *P* values > 0.10 for both; Extended Data Table 2). Heat showed different timing depending on outcome definition: the county-level composite DLNM was delayed, whereas the outcome-specific *E. coli* DLNM peaked at lag 0 and was significant only through lags 0–2 (Extended Data Table 6), consistent with different monitoring windows for composite versus microbial outcomes (see Discussion).

A counterfactual monitoring allocation analysis (Supplementary Tables 12–13, 16) showed that monthly precipitation and heat triggers lost most targeting value after calendar-month matching, but daily post-precipitation triggers captured 40.7% of upper-tail turbidity days (enrichment ratio = 2.77), consistent with submonthly follow-up being useful.

### Robustness and sensitivity analyses

Sensitivity analyses generally supported the main findings (Extended Data Tables 2, 4, 7; Supplementary Tables 2, 4, 5). Placebo tests were null (all *P* > 0.30), wild cluster bootstrap confirmed conventional inference, and excluding 2020 left coefficients unchanged. Both binary exposures showed monotone dose-response gradients (Extended Data Table 6). Service-area reassignment left all three headlines nearly unchanged (Supplementary Table 4), and Hydrologic Unit Code (HUC) 8 source-watershed reassignment strengthened the precipitation-turbidity estimate (Supplementary Table 14). An alternative HMS smoke panel reproduced the pooled association (Supplementary Table 5).

### Independent validation with measured water quality

**Figure 6 |:**
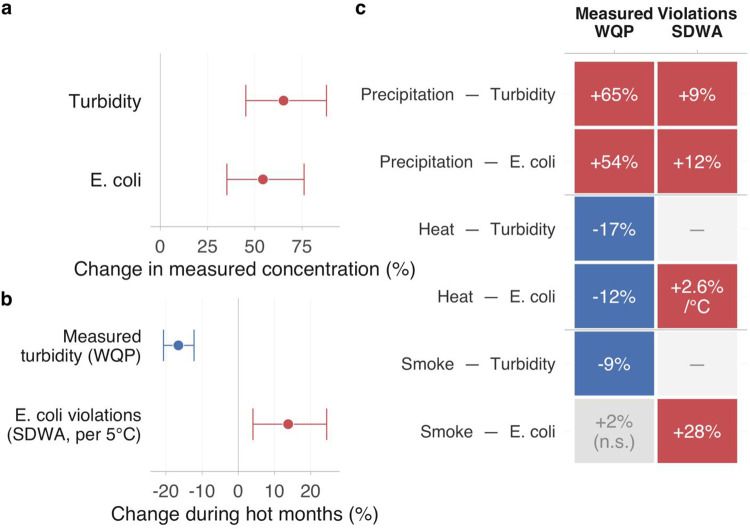
Independent water quality measurements corroborate violation-based findings and highlight differentiated hazard patterns. **a**, Change in measured turbidity and *E. coli* concentrations during extreme precipitation months (>county-specific P90) relative to normal months, from EPA/USGS Water Quality Portal drinking water monitoring sites (92,620 county-months, 1,141 counties). Dots show point estimates; horizontal bars show 95% confidence intervals. Both contaminants increase substantially, consistent with precipitation-driven water quality degradation. **b**, The heat paradox: hot months (>90th percentile) are associated with decreased measured turbidity (blue, consistent with reduced streamflow) but increased *E. coli* violation rates (red, from SDWA violation models), suggesting a pathway distinct from the precipitation signal and more compatible with operational stress than with broad degradation at monitored sites. This threshold-based WQP view is descriptive; the primary PWS heat estimand in the manuscript is continuous per °C. **c**, Summary heatmap comparing measured water quality changes (WQP, left) with regulatory violation changes (SDWA, right) across all three hazards. Red indicates increases, blue indicates decreases, grey indicates null. The divergent pattern for heat (blue measured concentrations, red violations) identifies a contrast from the precipitation pathway (red in both columns). WQP measurements include both source-water and finished-water samples (see Methods). All WQP models use county and year-month fixed effects with state-clustered standard errors.

Independent WQP validation (Figure 6; Extended Data Table 5) confirmed the hazard hierarchy. Monthly measured turbidity and *E. coli* both increased during extreme precipitation (65% and 54%; both *P* < 0.001), including in a treated-water-adjacent subset (Supplementary Table 15). Heat showed a scale-dependent paradox: monthly measured concentrations decreased during hot months, contrasting with the positive within-site daily shifts; a warm-day persistence probe sharpened the microbial signal (13.0% *E. coli* increase; *P* = 0.002; Supplementary Tables 17, 19). Smoke validation was weakest: monthly measured *E. coli* was null (*P* = 0.67), though a transport-plume event study and HMS plume replacement both showed a delayed far-transport signal (Supplementary Tables 16, 18). Lead and Copper Rule (LCR) tap-side checks were null (Supplementary Table 12).

Mechanism probes (Supplementary Tables 8, 9, 17) supported differentiated pathways: stream discharge mediated roughly half the precipitation-turbidity coefficient, outage intensity did not mediate heat or smoke effects, and no broad hazard-linked enforcement surges were detected.

## Discussion

Under a common national compliance framework, three climate hazards occupy sharply different evidentiary tiers: precipitation disruption is broad and robust, heat produces a narrower but reproducible microbial signal, and wildfire smoke remains identification-sensitive and exploratory. This graded hierarchy is the central finding, not any single hazard effect, because it reveals that climate-driven compliance vulnerability is pathway-specific rather than generic. For precipitation, multiple lines of evidence converge on a runoff/transport pathway: stream discharge mediates roughly half the precipitation-turbidity association (Supplementary Table 8), a hydrologically aligned HUC8 source-watershed reassignment strengthens the estimate relative to county-of-record linkage (Supplementary Table 19), the signal concentrates in surface-water systems and persists in a treated-water-adjacent WQP subset (Supplementary Table 20), and the population-weighted attenuation points to disproportionate structural vulnerability in smaller systems rather than a broad population-average burden. For heat, the evidence is more ambiguous. Monthly measured concentrations move in the opposite direction from violations, though the WQP site composition shifts substantially across temperature quintiles (the source-water fraction drops from ~97% in the coldest to ~49% in the warmest quintile), so part of that monthly divergence may reflect changing sample composition rather than a true mechanistic contrast. (The composition check was conducted for temperature; analogous shifts during precipitation events are less likely because the daily within-site models, which hold site composition fixed, confirm the same direction and larger magnitudes.) Within-site daily models show short-run microbial increases, the association is present in groundwater but absent in surface systems (Supplementary Table 5), and a warm-day persistence probe sharpens the microbial signal while threshold-defined hot months do not reproduce it (Supplementary Tables 15, 22, 24). These pieces collectively argue against broad upstream deterioration but do not isolate a single operational pathway; the current data cannot distinguish treatment-plant stress, distribution-system conditions, sampling composition, or reporting structure. For smoke, the county-month WQP validation is weak and source-type evidence is mixed, so any deposition-based interpretation should remain provisional.

The smoke findings require the most careful interpretation. The pooled association attenuates to null under strict seasonal controls, consistent with seasonal confounding. The transport identification (200–1,000 km from active fires) addresses this by isolating atmospheric transport from local fire confounders, but neither the distance gradient nor the weather-controlled restoration fully rules out residual seasonal co-occurrence, and the pattern could reflect suppression bias from conditioning on seasonal mediators rather than genuine confounding control. Two alternative explanations received direct empirical attention: monitoring disruption near fires was not supported by the WQP sampling-intensity check (Supplementary Table 4), and grid disruption was not supported by the EAGLE-I outage analysis (Supplementary Table 9). The most persuasive smoke evidence is the convergence of two independent transport-only timing designs: both the county smoke proxy and direct HMS plume polygons show a delayed (lag-4) far-transport *E. coli* signal in the 500–1,000 km band with no same-day effect (Supplementary Tables 21, 23), a pattern more compatible with delayed atmospheric deposition than with local-fire disruption. We nevertheless regard smoke as an exploratory transport probe rather than a co-equal national pathway. Statistical power is limited in this sub-sample: the transport zone retains only 90.4% of the *E. coli* estimation sample (Supplementary Table 26), and the pooled transport estimate already attenuates to marginal significance under strict seasonal controls. The exploratory demographic gradients (Extended Data Figure 2c–d) rely on fragile few-cluster models and should be interpreted only as hypothesis-generating.

Timing analyses offer a more actionable guide for adaptation. Smoke and precipitation signals are concentrated in the exposure month, whereas the *E. coli*-specific heat DLNM peaks at lag 0 (Extended Data Table 6), suggesting different monitoring windows. For outcomes assessed against running annual averages (e.g., disinfection byproducts), same-month associations may reflect cumulative antecedent conditions; restricting to genuinely monthly-sampled outcomes preserves the main precipitation findings.

Several limitations most constrain interpretation. First, detection bias operates asymmetrically: the Surface Water Treatment Rule mandates increased monitoring during precipitation events, potentially inflating the precipitation signal, while smoke and heat events may disrupt routine monitoring, biasing those associations toward the null. The WQP validation suite and site-visit panel (Supplementary Table 17) substantially reduce but do not eliminate this concern, and we accordingly characterise all findings as violation risk (regulatory noncompliance) rather than contamination per se. Second, exposures are assigned at the county-month level, introducing measurement error that is non-classical for binary thresholds and directional for surface systems drawing from remote watersheds; service-area and HUC8 reassignment sensitivities (Supplementary Tables 6, 19) are reassuring but do not recover true intake locations. Third, all associations are ecological and cannot establish individual-level risk; time-varying confounders such as enforcement regime changes and infrastructure investment cycles are not absorbed by the fixed effects structure. Fourth, approximately 43 million Americans relying on private wells fall outside SDWA entirely, and Tribal community water systems, though included in SDWIS, are not separately identifiable; these omissions limit generalisability to some of the communities most likely to experience compounding disadvantage.

These findings argue for restructuring drinking water oversight around climate-specific risk rather than static compliance schedules, but the operational case is not equally strong across hazards. The clearest evidence supports short-window post-precipitation follow-up for turbidity and coliform, concentrated in smaller systems. The case for heat-triggered microbial follow-up is narrower, because the compliance signal is reproducible but the mechanism unresolved. The case for transported-smoke attention is narrower still, resting on a targeted transport-zone probe rather than a broadly established trigger. Current SDWA monitoring schedules are calendar-based and hazard-blind; climate-responsive triggers could detect compliance failures that fixed schedules miss, but the useful trigger horizon differs by hazard and is strongest for precipitation at submonthly timescales. Implementing such changes requires confronting structural inequities: small community water systems constitute 88% of the regulated universe but have the least capacity to absorb additional monitoring burdens. Mandating more frequent sampling without corresponding financial and technical support risks documenting disparities rather than resolving them. At daily resolution, top-decile precipitation days captured 40.7% of upper-tail turbidity days at monitored sites; translating that enrichment into operational triggers for smaller systems is the most concrete policy implication of these findings. More broadly, the graded hierarchy reported here suggests that climate adaptation for drinking water should not be pursued as a single generic programme but as a set of hazard-specific responses calibrated to the strength of the underlying evidence.

## Methods

### Data sources

#### Water system violations.

We obtained all Safe Drinking Water Act violations from the EPA’s Safe Drinking Water Information System (SDWIS) for the period 2006–2020. Violations were classified into twelve outcome categories: turbidity, total coliform, *E. coli*, disinfection byproducts (trihalomethanes [THMs] and haloacetic acids [HAA5]), treatment technique, surface water treatment rule, monitoring and reporting, health-based, nitrate, lead and copper, arsenic, and total organic carbon (TOC). We retained community water systems whose SDWIS operating records overlapped the study window, using FIRST_REPORTED_DATE and PWS_DEACTIVATION_DATE (with LAST_REPORTED_DATE fallback only when inactive systems lacked a deactivation date), and constructed system-months only within that overlap. Violation indicators were then expanded across the full recorded SDWIS compliance period from COMPL_PER_BEGIN_DATE to COMPL_PER_END_DATE, truncated to the study window, and aggregated to the public water system × month level. For regulatory-activity checks, we additionally obtained SDWIS site-visit records, including visit reasons and evaluation codes used to identify sanitary surveys, enforcement or investigation visits, and visits with significant deficiencies, and aggregated those records to the same PWS-month panel. Restricting to systems in counties covered by the contiguous-US smoke and climate products yielded 9,021,476 climate-linked system-month observations across 56,351 community water systems. Tribal water systems under EPA direct implementation are included in SDWIS but represent a small fraction of the sample; their results are not reported separately due to limited statistical power. *E. coli* violations are confirmed under the Total Coliform Rule and overlap substantially with total coliform violations; these two outcomes should be interpreted as a single microbial signal in the Results rather than as independent mechanistic tests. Monitoring frequencies under SDWA vary by rule: the Total Coliform Rule requires monthly sampling for most systems, whereas inorganic contaminants (arsenic, nitrate) may be monitored annually or triennially, and disinfection byproduct compliance is assessed against running annual averages. Consequently, the effective estimation sample varies substantially across outcome categories, reflecting rule applicability, reporting frequency, the length of recorded compliance periods, and the separation of never-violating units in fixed-effects estimation (see Statistical analysis). Accordingly, Figure 2 is interpreted as a broad screening panel with overlapping and differently timed endpoints, while the main narrative emphasises the more comparable microbial and operational outcomes.

#### Wildfire smoke.

Daily county-level wildfire smoke PM_2.5_ concentrations were obtained from the Childs et al.^14^ machine learning prediction model, which combines satellite aerosol optical depth, chemical transport model output, and ground-based monitoring to isolate smoke-specific PM_2.5_ from total ambient concentrations at the county level across the contiguous US (2006–2020). We aggregated daily estimates to county-months, defining smoke exposure as ≥10 smoke days per month (primary) with 5-day and 15-day thresholds as sensitivity analyses.

#### Temperature.

Daily maximum temperature (T_max_) was obtained from PRISM at the county level. For county-level analyses, we used a distributed lag non-linear model framework evaluated at high percentiles of the local temperature distribution. For PWS-level analyses, the primary heat exposure is continuous monthly mean T_max_ (per °C), which preserves within-system variation under fixed effects; threshold-defined hot-month indicators are used only in auxiliary validation and sensitivity analyses. Accordingly, the main heat estimand is interpreted as a monthly heat-intensity sensitivity rather than as a threshold-defined extreme-event effect.

#### Precipitation.

Monthly total precipitation from PRISM was used both as a control variable in smoke and heat models and as a primary exposure in Design 3. Extreme precipitation months were defined as those exceeding the county-specific 90th percentile of the 2006–2020 distribution, with 80th and 95th percentile thresholds, county × month-of-year-specific P90 thresholds (to address seasonality), and standardized precipitation anomalies (z-scores relative to county × month climatology) as sensitivity analyses.

#### Fire perimeters.

The Monitoring Trends in Burn Severity (MTBS) dataset provided 30,730 fire perimeter polygons (1984–2022) with attributes including fire type, ignition date, and burned area. Fire perimeters were intersected with Hydrologic Unit Code (HUC)-8 watershed boundaries from the USGS Watershed Boundary Dataset to compute watershed-level burned area. For the transport distance analysis, each county-month was assigned the minimum haversine distance from the county centroid to any MTBS burn-boundary centroid with an ignition date in the same or preceding calendar month.

#### Water system characteristics and demographics.

PWS characteristics (water source type, system size, ownership, population served) were obtained from SDWIS (Supplementary Table 2). All gridded exposure products (PRISM temperature and precipitation at ~4 km, Childs et al. smoke PM_2.5_ at ~10 km) are area-weighted to the county level and then linked to individual PWSs via the county FIPS code of record in SDWIS. This county-level assignment means that the unit of exposure variation is the county-month, not the individual system; systems whose service areas span multiple counties or draw from out-of-county sources will have exposure measurement error, which would generally attenuate estimates toward the null. As a more hydrologically aligned sensitivity for surface systems, we additionally linked PWSs to their assigned HUC8 source watersheds and rebuilt precipitation exposures at the HUC8-month scale for a matched source-watershed comparison (Supplementary Table 19). County-level demographic variables (poverty rate, minority percentage, median household income) were obtained from the American Community Survey 5-year estimates. We used the most recent ACS cross-section available in the processed county file and treated these measures as time-invariant ecological stratifiers rather than as time-varying county covariates.

### Exposure definitions

**Extended Data Figure 1 |:**
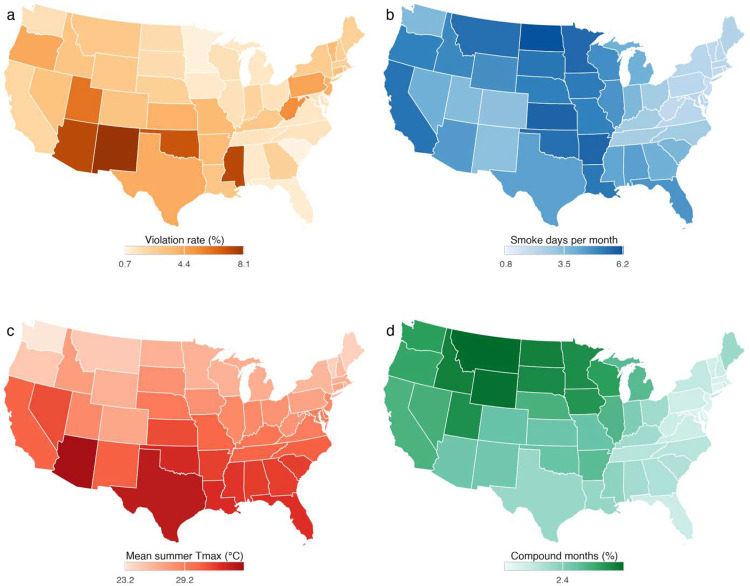
Geographic distribution of exposures and violations. **a**, Mean monthly violation rate per 10,000 system-months by state, showing baseline geographic heterogeneity in regulatory noncompliance. **b**, Mean wildfire smoke days per month (2006–2020). **c**, Mean summer daily maximum temperature. **d**, Fraction of county-months with compound hazard co-occurrence.

The primary exposure definitions are as follows. Wildfire smoke: ≥10 county-level smoke days per month (Childs et al. smoke PM_2.5_; sensitivity at 5 and 15 days). Heat (county-level): DLNM cross-basis with natural cubic spline and 0–6 month lags (PRISM T_max_; sensitivity at 75th and 99th percentile references). Heat (PWS-level): continuous monthly mean T_max_ per °C. Extreme precipitation: county-month exceeding the county-specific 90th percentile (PRISM; sensitivity at 80th, 95th, and county × month-of-year-specific P90 thresholds). Compound: four-category county-month classifications (Neither, hazard A only, hazard B only, Both), applied to smoke × heat as the primary compound diagnostic and extended exploratorily to smoke × precipitation and precipitation × heat. All exposures are assigned at the county level and linked to public water systems via county FIPS (Extended Data Figure 1 maps the geographic distribution of exposures and violations).

We distinguish three evidence tiers in reporting. Primary analyses are the 12-outcome screening panel and the hazard-specific pathway models that anchor the main text, with precipitation-turbidity carrying the greatest interpretive weight because it is the broadest and most consistently validated result. Secondary analyses are validation and misclassification checks (WQP, NWIS, EAGLE-I, service-area overlap, HMS smoke, and LCR).

Exploratory analyses include the burn-scar pathway, regional and environmental justice heterogeneity, and the counterfactual monitoring allocation exercises. The smoke transport-distance models function as a targeted identifying probe prompted by attenuation in the pooled smoke model and are interpreted accordingly as exploratory rather than confirmatory.

### Statistical analysis

#### Design 1: Wildfire smoke.

We estimated the effect of smoke exposure on violation counts using two-way fixed effects (TWFE) Poisson regression (fixest::fepois), with PWS fixed effects and year-month fixed effects. The model specification was:

$$\text{log}\left(\mu_{it}\right)=\alpha_{i}+\gamma_{i}+\beta \cdot {\text{Smoke}}_{ct}$$

where *μ*_*it*_ is the expected violation count for system *i* in month *t, α*_*i*_ are system fixed effects, *γ*_*t*_ are year-month fixed effects, and Smoke_ct_ is the binary smoke indicator for county *c* in month *t*. The baseline specification does not include weather covariates; precipitation and temperature controls are added in the seasonal control ladder (see below) to assess robustness to weather confounding. System fixed effects absorb time-invariant confounders (water source type, infrastructure age, system capacity, community demographics), while year-month fixed effects absorb national-level temporal trends in regulatory enforcement, reporting practices, and climate conditions. This TWFE specification is used here for a recurrent, time-varying environmental exposure rather than a one-time absorbing treatment. More generally, de Chaisemartin and D’Haultfœuille^15^ show that TWFE can produce negative weighting of heterogeneous treatment effects; dose-response and placebo analyses (Extended Data Table 2) assess this concern.

Two spatial scales are used in the analysis. County-level models are used for Design 1 (smoke–turbidity) and Design 2 (heat DLNM) because the national linkage between climate exposures, fire distance metrics, and violation outcomes is harmonized at the county-month level, and the DLNM framework requires a single repeated time series within each unit. The underlying exposure products are available at finer native resolution (for example, daily gridded smoke PM_2.5_ and gridded meteorological fields), but nationally consistent public data on intake location, service area, and sampling timing are insufficient to support finer-scale assignment across all systems in the study. PWS-level models are the primary specification for pathway-specific outcomes (including the twelve-category analysis in Figure 2), linking county-level exposure to system-level violation counts via county FIPS. The PWS-level specification is preferred because it isolates individual contaminant violations and uses within-system variation; county-level composite-outcome analyses provide a complementary cross-check using different sources of identifying variation.

At the PWS level, violation indicators are binary (0/1 for any violation of each type in a system-month); applying Poisson regression to binary outcomes estimates the log-relative risk, with cluster-robust standard errors providing valid inference under this quasi-likelihood interpretation^16^. County-level DLNM outcomes are violation counts (number of systems with a violation in that county-month), which follow a count distribution and are modelled with standard Poisson regression. In fixed-effects Poisson estimation (fixest::fepois), systems or county-months with all-zero outcomes across the panel are automatically separated from the estimation sample because their fixed-effect coefficient diverges to negative infinity; this affects the reported N but does not bias the remaining coefficients^17^. The estimand for separated outcomes is therefore the treatment effect among ever-violating systems, those with at least one violation during the panel, rather than a population-average effect across all community water systems. Consequently, the effective sample size varies across outcome categories (from ~700,000 system-months for *E. coli* to >7 million for monitoring violations), reflecting both differences in rule applicability and the separation of never-violating units (Supplementary Table 26).

Standard errors in PWS-level models were clustered at the state level to account for spatial correlation in both climate exposure and regulatory enforcement regimes. State-level clustering reflects the administrative structure of SDWA enforcement, in which state primacy agencies conduct the majority of compliance monitoring. County-level models (the DLNM and compound interaction analyses) use county-clustered standard errors because the unit of observation is the county-month. Because the effective number of clusters varies modestly across specifications after fixed-effects Poisson separation, we supplement conventional cluster-robust inference with a score-based wild cluster bootstrap using Rademacher weights for the three headline results (Extended Data Table 2), which provides more reliable rejection rates when the number of clusters is modest^18^. The bootstrap was not extended to the division-level EJ analyses, where cluster counts are as low as 3–4 states per division.

#### Design 2: Heat.

County-level heat effects were estimated using distributed lag non-linear models (DLNM) with a cross-basis of monthly temperature and lag (0–6 months), using a natural cubic spline basis for both temperature and lag dimensions and county, year, and month-of-year fixed effects with county-clustered standard errors; the county-level DLNM controls for concurrent smoke days, which could introduce post-treatment bias for outcomes also affected by smoke, though the magnitude is likely small given the modest smoke effects. At the PWS level, we used continuous monthly mean T_max_ in the TWFE Poisson specification. As a post hoc mechanism probe, we additionally stratified the heat-*E. coli* model by main water source type (surface vs groundwater) and estimated a direct heat × surface interaction.

#### Design 3: Extreme precipitation.

We estimated the effect of extreme precipitation on violation counts using the same TWFE Poisson specification as Design 1, replacing the smoke indicator with a binary extreme precipitation indicator (county-month precipitation > county-specific 90th percentile). We stratified precipitation models by water source type (surface vs groundwater), ownership (public vs private), and system size (<=3,300 vs >3,300 population). To parallel the main-text operational-targeting analysis, we also fit post hoc stratified versions of the smoke-*E. coli* and heat-*E. coli* models by ownership and system size, and estimated selected interaction terms (smoke × ownership, heat × source type, heat × system size, precipitation × source type, precipitation × ownership). Stratified contrasts without direct interaction support are interpreted descriptively rather than as confirmed effect modification.

#### Design 4: Compound interaction.

We estimated four-category county-month exposure models (Neither, hazard A only, hazard B only, Both) and computed the Relative Excess Risk due to Interaction (RERI) as:

$$\text{RERI}={\text{RR}}_{\text{Both}}-{\text{RR}}_{\text{Smoke}}-{\text{RR}}_{\text{Heat}}+1$$

Negative RERI indicates sub-additivity; positive RERI indicates super-additivity. The primary compound model paired smoke with extreme heat; parallel exploratory models paired smoke with extreme precipitation and extreme precipitation with extreme heat. For smoke × heat, the reference group (“Neither”) comprises months with <10 smoke days and no extreme-heat month; analogous definitions were used for the other pairings. Because the vast majority of county-months fall into the reference category over a 15-year period, “Both” captures relatively uncommon co-occurring hazard months rather than catastrophic compound extremes; the RERI should be interpreted accordingly.

#### Design 5: Sequential burn scar (exploratory).

For the watershed analysis subset, we assigned systems with valid ZIP-to-HUC-8 matches to HUC-8 watersheds via ZIP code centroids. Trailing 24-month burned area within each system’s watershed was computed from MTBS fire perimeters. We estimated the interaction between burn scar presence and high precipitation on violation counts using the TWFE Poisson specification at the PWS × month level. This analysis is reported in Extended Data Note 1; we consider it exploratory because statistical power is limited by the small number of systems with watershed-level fire exposure.

#### Regional stratification and demographic heterogeneity.

We stratified main effects by nine Census divisions and tested for demographic effect modification by interacting wildfire smoke exposure with county-level minority percentage and poverty rate (both scaled per 10 percentage points) across all divisions and nationally (20 interaction tests). We applied the Benjamini–Hochberg procedure across all 20 tests and report FDR-adjusted q-values. For divisions with significant interactions, we probed mechanisms by stratifying by system ownership (public vs private), water source type (surface vs groundwater), and system size (≤3,300 vs >3,300 population served). All interactions are ecological (county-level demographic characteristics interacted with county-level exposure) and cannot be interpreted as individual-level effect modification.

#### Multiple testing.

The unified 12-outcome × 3-exposure analysis (Figure 2) involves 36 screened exposure-outcome combinations. These should not be interpreted as 36 independent mechanistic hypotheses because some outcomes overlap structurally (*E. coli*/coliform; health-based composites) and some are monitored on slower cadences than the monthly microbial outcomes. We designate microbial outcomes (*E. coli*/coliform and turbidity) as primary and the remaining nine categories as secondary. To formally assess the false discovery rate across this broad screening panel, we apply the Benjamini–Hochberg procedure to all 36 tests and report both unadjusted *P* values and FDR-adjusted q-values.

#### Robustness checks.

##### Exposure definition sensitivity.

Analyses included alternative smoke thresholds (5 and 15 days), continuous smoke-day dose–response models, month-specific smoke definitions (county × month-of-year-specific P90 thresholds and standardized smoke anomalies), exclusion of extreme fire years (2017, 2018, 2020), distributed lag models entering contemporaneous and 1- and 2-month lagged exposure jointly (*t* + *t* − 1 + *t* − 2) for smoke and precipitation, population-served-weighted models (to test whether results are driven by small systems), and threshold-defined hot-month indicators (P90 and P95 of county-specific T_max_) in the PWS-level heat-*E. coli* model.

##### Temporal controls.

Alternative time fixed effects (state × year; census region × month-of-year; region × month + region × year), placebo tests using 6-month-forward (future) exposure, and exclusion of 2020 (to assess COVID-19 monitoring disruption).

##### Spatial assignment.

To assess exposure misclassification from county-of-record linkage, we repeated the headline models in the subset of systems with public EPA service-area boundaries, comparing the default county-of-record assignment with both a representative-point proxy and true area-weighted county overlap (Supplementary Table 6). We then added a more hydrologically aligned intake-oriented sensitivity for surface systems by linking eligible PWSs to assigned HUC8 source watersheds and comparing county- versus HUC8-assigned precipitation models on the same sample (Supplementary Table 19).

##### Alternative exposure measurement.

We built an alternative county-month smoke-day panel from NOAA Hazard Mapping System daily smoke polygons and repeated paired smoke models under both the Childs and HMS exposure definitions (Supplementary Table 7).

##### Monitoring and regulatory activity checks.

To probe whether local-fire emergencies suppressed independent monitoring, we regressed county-month WQP sampling counts on smoke distance bins (Supplementary Table 4). To distinguish changing contamination from changing regulatory activity, we estimated hazard associations for site visits, sanitary surveys, and enforcement visits in 0–1 month windows (Supplementary Table 17).

##### Policy translation.

A counterfactual monitoring allocation analysis compared observed hazard-triggered monitoring windows with equal-budget hazard-blind allocation within systems and within systems × calendar month; precipitation used same-month triggers, heat used the hot month plus the next two months (matching the *E. coli* lag window), and smoke used the transported-smoke zone (Supplementary Table 12). We repeated the stricter month-matched comparison within subgroups defined by system size, ownership, source type, and cumulative prior significant-deficiency history. A daily WQP counterfactual analysis compared site-specific top-decile hazard triggers with upper-tail daily turbidity outcomes (Supplementary Table 13).

##### Negative control framework.

The outcome decomposition across twelve violation categories (Figure 2) provides a broad screening contrast in the spirit of the negative control outcome framework^19^: chemical contaminants (lead and copper, nitrate) have less plausible acute-event pathways for the exposures studied, and their null associations support the specificity of the microbial and operational effects, though we cannot rule out all possible pathways for these outcomes.

#### Seasonal control ladder.

To assess differential robustness across hazards, we applied a uniform sequence of progressively stricter seasonal fixed effects to all three hazard–outcome pairs: (i) PWS + year-month (absorbing national temporal trends), (ii) PWS + year + month-of-year (separating annual trends from a national seasonal cycle), (iii) PWS + year + region × month-of-year (allowing the seasonal cycle to vary by census region), (iv) PWS + year + state × month-of-year (allowing state-specific seasonal cycles), (v) PWS + year + county × month-of-year (absorbing county-specific seasonality, the strictest purely temporal control), and (vi) specification (v) plus concurrent weather controls (temperature and precipitation for smoke; temperature only for precipitation; precipitation only for heat). Throughout, “county × month-of-year” denotes the interaction of county identity with calendar month (January–December), not county × calendar date; this absorbs county-specific seasonal patterns while preserving year-to-year variation for identification.

#### Transport smoke identification.

To separate transported wildfire smoke from local fire confounders, we computed haversine distances from each county centroid to all active MTBS burn-boundary centroids in each county-month and classified smoke exposure into distance bins (0–100, 100–200, 200–500, 500–1,000, and >1,000 km). We defined a transport zone (200–1,000 km from the nearest active fire centroid) where counties receive meaningful smoke PM_2.5_ by atmospheric transport without local fire effects (ash runoff, emergency responses, infrastructure disruption). The transport identification was tested under the full seasonal control ladder described above. Alternative distance cutpoints (150/300 km lower bound; 800/1,200 km upper bound) were tested to assess sensitivity to the boundary definitions (Extended Data Table 6).

#### Water Quality Portal validation.

##### Monthly county-level validation.

To test whether violation-based findings reflect genuine water quality degradation rather than monitoring or enforcement artefacts, we obtained measured turbidity and *E. coli* concentrations from the EPA/USGS Water Quality Portal (waterqualitydata.us), restricting to drinking water monitoring sites during the study period (2006–2020). Measurements were aggregated to county-month medians and merged with the climate exposure panel. WQP measurements include both source-water (ambient monitoring) and finished-water (compliance) samples; the primary monthly validation therefore captures a combined signal of source water quality and treatment effectiveness and cannot by itself isolate treatment-plant from source-water mechanisms. We regressed log-transformed concentrations on the same exposure variables using county and year-month fixed effects with state-clustered standard errors.

##### Daily within-site sharpening.

To sharpen temporal identification, we estimated daily within-site case-crossover-style models on site-day WQP panels with site and year-month fixed effects, focusing on same-day precipitation, a 0–7 d precipitation window for turbidity, a 0–14 d heat window, and a lag-4 smoke window for *E. coli* (Supplementary Table 11). We stratified daily precipitation models by flowing versus standing hydro-class as a proxy for source-water-likeness (Supplementary Table 10). To sharpen the smoke interpretation, we restricted the daily *E. coli* site-day panel to transported-smoke months in the 200–500 km and 500–1,000 km fire-distance bands, estimating within-site × month smoke-intensity models at same-day and lag-4 alignments (Supplementary Table 21), then replaced the county smoke proxy with direct daily NOAA HMS plume polygons at the site-day level (Supplementary Table 23). To test whether heat acts through sustained warm periods rather than isolated hot days, we reparameterized daily site weather into rolling counts of warm days above site-specific 75th–90th percentile thresholds over the prior 7 and 14 days (Supplementary Table 22), and examined same-day heatwave-day indicators and outage-vulnerability heat checks as supplementary probes (Supplementary Table 24).

##### Treated-water and tap-side checks.

To move the monthly validation closer to treated water, we isolated the narrow subset of WQP turbidity sites tagged as public-water-supply facilities or distribution/pipe locations and repeated the monthly hazard models in that subset (Supplementary Table 20). As a direct tap-side supplement, we aggregated national SDWIS Lead and Copper Rule customer-tap samples to PWS monitoring-period 90th percentiles for lead and copper and estimated PWS- and year-month-fixed-effects models for log 90th percentiles and lead action-level exceedance (Supplementary Table 14); because those monitoring periods are often multiyear, we treat that check as a coarse complement rather than a short-window hazard test.

##### Mechanism probes.

We linked daily USGS National Water Information System (NWIS) discharge observations to WQP monitoring sites with USGS station identifiers, then estimated models of log turbidity/*E. coli* on daily weather with and without log discharge adjustment to test runoff mediation (Supplementary Table 8). As an operational-disruption probe, we aggregated county-level EAGLE-I outage observations to county-months for 2014–2020 and tested whether outage intensity mediated the heat-*E. coli* and transport-smoke-*E. coli* associations (Supplementary Table 9).

## Extended Data

Extended Data Note 1: Exploratory burn scar analysis

We examined a sequential pathway from wildfire to burn scar formation to precipitation-driven contamination by linking MTBS fire perimeters to HUC-8 watershed boundaries and assigning public water systems to their source watersheds. Systems whose watersheds experienced wildfire in the preceding 24 months showed a suggestive increase in turbidity violations during high-precipitation months (RR = 1.038; *P* = 0.09). In a complementary county-level analysis, point estimates strengthened when restricting to increasingly severe fires: all MTBS fires (*P* = 0.09), wildfire only (*P* = 0.08), and large wildfires exceeding 10,000 acres (*P* = 0.06; Supplementary Figure 2).

However, this pathway did not achieve conventional significance in any specification, and robustness checks were uniformly null: alternative temporal windows (12-month, *P* = 0.60; 36-month, *P* = 0.37), coliform as outcome (*P* = 0.997), drought month exclusion (*P* = 0.95), and restriction to western states (*P* = 0.63) all failed to detect an effect (Supplementary Table 1). The most likely explanation is insufficient statistical power: only a small fraction of systems have watersheds with recent fire exposure, and the burn scar × precipitation interaction further dilutes the effective sample.

A three-way interaction with community poverty (RR = 1.070; *P* = 0.04) was the only specification to achieve significance, but we interpret this cautiously given the null main effect on which it is conditioned. With multiple specifications tested (five robustness checks, two alternative outcomes, and several EJ interactions), a single significant three-way interaction is consistent with the expected false positive rate and should not be treated as confirmatory evidence without independent replication.

Nominal inverse associations between burn scar exposure and arsenic (RR = 0.891; *P* = 0.02) and nitrate violations (RR = 0.828; *P* = 0.02) did not survive diagnostic scrutiny. A placebo test using future fire (24–48 months later) as exposure produced a similarly inverse nitrate estimate (RR = 0.831; *P* = 0.009), indicating spatial confounding: watersheds that eventually burn tend to have fewer agricultural violations for reasons unrelated to fire. The arsenic association was absent in western states where the mechanism should operate (RR = 0.942; *P* = 0.35) and showed no dose–response with continuous burned area (RR = 0.990; *P* = 0.16).

We report these results transparently as a foundation for future work. Higher-resolution exposure data (watershed-specific rather than HUC-8), longer follow-up periods capturing additional fire seasons, and focus on specific high-fire regions may provide the statistical power needed to test this pathway rigorously.

**Extended Data Figure 2 |:**
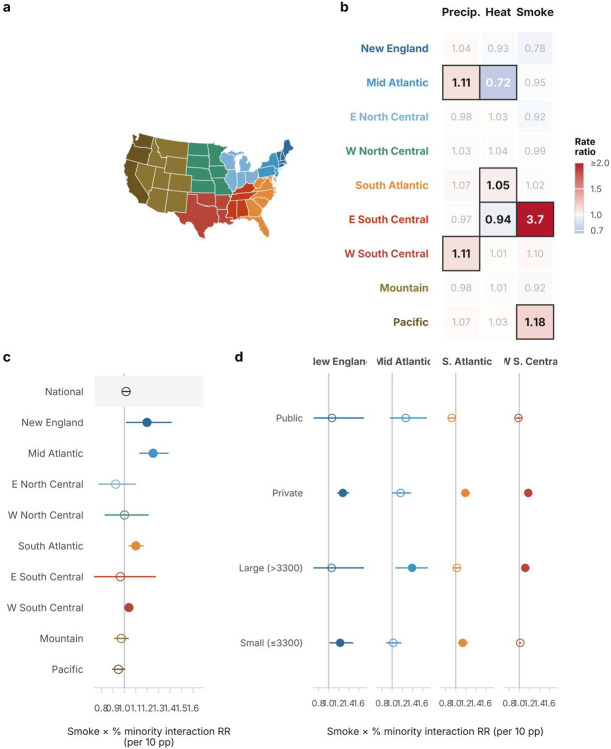
Exploratory regional heterogeneity and demographic effect modification of wildfire smoke. **a**, US map of nine Census divisions with colors shared across all panels. **b**, Main effect rate ratios for the three hazards by Census division. Bold tiles with dark borders indicate *P* < 0.05; faded tiles indicate *P* ≥ 0.05. Precipitation disruption is the most geographically uniform; heat and smoke effects are regionally variable. **c**, Interaction rate ratios for wildfire smoke × county-level minority percentage (per 10 percentage-point increase) on *E. coli* violations, by division and nationally. Filled circles indicate *P* < 0.05; hollow circles indicate *P* ≥ 0.05. FDR-significant interactions appear in the Mid Atlantic, South Atlantic, and W South Central divisions; poverty interactions were generally null except for a nominally significant E South Central estimate that relies on only 4 state clusters (Supplementary Table 3). **d**, Mechanism probes for the smoke × minority interaction in the four divisions with nominally significant interactions, stratified by system ownership and size. These panels are hypothesis-generating and several division estimates rely on few-cluster inference. All models use PWS and year-month fixed effects with state-clustered standard errors.

**Table:** 

Outcome	Exposure	RR	SE	P value	N
Nitrate	Wildfire smoke	1.036	0.037	0.333	1,427,938
Nitrate	Extreme heat	1.001	0.003	0.581	1,427,938
Nitrate	Extreme precipitation	1.015	0.019	0.439	1,427,938
Lead/Copper	Wildfire smoke	1.046	0.047	0.345	4,065,461
Lead/Copper	Extreme heat	1.008	0.009	0.332	4,065,461
Lead/Copper	Extreme precipitation	1.058	0.037	0.124	4,065,461
E. coli	Wildfire smoke	1.098	0.041	0.023	676,861
E. coli	Extreme heat	1.026	0.009	0.004	676,861
E. coli	Extreme precipitation	1.137	0.037	< 0.001	676,861
SWTR	Wildfire smoke	1.005	0.022	0.835	5,387,169
SWTR	Extreme heat	1.004	0.002	0.063	5,387,169
SWTR	Extreme precipitation	1.101	0.018	< 0.001	5,387,169
TOC	Wildfire smoke	0.819	0.041	< 0.001	181,706
TOC	Extreme heat	1.009	0.008	0.247	181,706
TOC	Extreme precipitation	1.022	0.025	0.395	181,706
Arsenic	Wildfire smoke	1.018	0.024	0.453	889,306
Arsenic	Extreme heat	1.000	0.002	0.897	889,306
Arsenic	Extreme precipitation	1.013	0.015	0.373	889,306

**Table:** 

Panel	Analysis	Term	RR	95% CI	P value
A. Distributed lags	Smoke -> E. coli (t only)	smoke_10d	1.098	(1.013, 1.189)	0.023
A. Distributed lags	Smoke -> E. coli (t + L1)	smoke_10d	1.089	(1.006, 1.179)	0.035
A. Distributed lags	Smoke -> E. coli (t + L1)	smoke_10d_lag1	1.035	(0.956, 1.119)	0.397
A. Distributed lags	Smoke -> E. coli (t + L1 + L2)	smoke_10d	1.088	(1.005, 1.179)	0.038
A. Distributed lags	Smoke -> E. coli (t + L1 + L2)	smoke_10d_lag1	1.038	(0.958, 1.125)	0.361
A. Distributed lags	Smoke -> E. coli (t + L1 + L2)	smoke_10d_lag2	0.986	(0.901, 1.078)	0.758
A. Distributed lags	Precip -> E. coli (t only)	extreme_precip	1.137	(1.057, 1.223)	< 0.001
A. Distributed lags	Precip -> E. coli (t + L1)	extreme_precip	1.131	(1.053, 1.214)	< 0.001
A. Distributed lags	Precip -> E. coli (t + L1)	extreme_precip_lag1	1.062	(0.969, 1.164)	0.200
A. Distributed lags	Precip -> E. coli (t + L1 + L2)	extreme_precip	1.131	(1.053, 1.215)	< 0.001
A. Distributed lags	Precip -> E. coli (t + L1 + L2)	extreme_precip_lag1	1.059	(0.966, 1.162)	0.222
A. Distributed lags	Precip -> E. coli (t + L1 + L2)	extreme_precip_lag2	1.028	(0.960, 1.102)	0.426
B. Dose-response (smoke)	E. coli Linear	smoke_days	1.0104	(1.0041, 1.0167)	0.001
B. Dose-response (smoke)	E. coli Quadratic	smoke_days	1.0217	(1.0093, 1.0342)	< 0.001
B. Dose-response (smoke)	E. coli Quadratic	smoke_days_squared	0.9995	(0.9988, 1.0001)	0.115
B. Dose-response (smoke)	Turbidity Linear	smoke_days	0.9999	(0.9962, 1.0037)	0.976
B. Dose-response (smoke)	Turbidity Quadratic	smoke_days	1.0020	(0.9962, 1.0079)	0.494
B. Dose-response (smoke)	Turbidity Quadratic	smoke_days_squared	0.9999	(0.9997, 1.0001)	0.273
B. Dose-response (smoke)	Coliform Linear	smoke_days	1.0104	(1.0041, 1.0168)	0.001
B. Dose-response (smoke)	Coliform Quadratic	smoke_days	1.0222	(1.0098, 1.0348)	< 0.001
B. Dose-response (smoke)	Coliform Quadratic	smoke_days_squared	0.9994	(0.9988, 1.0001)	0.104
B. Dose-response (precip)	Turbidity per 10mm	mean_ppt_10mm	1.0066	(1.0049, 1.0083)	< 0.001
B. Dose-response (precip)	Coliform per 10mm	mean_ppt_10mm	1.0028	(0.9959, 1.0098)	0.422
B. Dose-response (precip)	DBP per 10mm	mean_ppt_10mm	1.0025	(1.0008, 1.0042)	0.004
B. Dose-response (precip)	Health-based per 10mm	mean_ppt_10mm	1.0036	(1.0022, 1.0049)	< 0.001
C. Alternative FE	E. coli ~ smoke_10d	pws_id + region × month	1.254	(1.094, 1.438)	0.001
C. Alternative FE	E. coli ~ smoke_10d	pws_id + ym_num (baseline)	1.098	(1.013, 1.189)	0.023
C. Alternative FE	Turbidity ~ extreme_precip	pws_id + region × month	1.011	(0.968, 1.057)	0.617
C. Alternative FE	Turbidity ~ extreme_precip	pws_id + ym_num (baseline)	1.083	(1.047, 1.121)	< 0.001
C. Alternative FE	E. coli ~ smoke_10d	pws_id + region × month + region × year	1.073	(0.978, 1.178)	0.137
C. Alternative FE	Turbidity ~ extreme_precip	pws_id + region × month + region × year	1.067	(1.029, 1.105)	< 0.001
D. Placebo tests	E. coli ~ smoke_10d_lead6	placebo	0.943	(0.790, 1.127)	0.521
D. Placebo tests	E. coli ~ smoke_10d	reference	1.122	(1.032, 1.219)	0.007
D. Placebo tests	Turbidity ~ extreme_precip_lead6	placebo	1.028	(1.000, 1.056)	0.050
D. Placebo tests	Turbidity ~ extreme_precip	reference	1.083	(1.047, 1.121)	< 0.001
D. Placebo tests	Coliform ~ extreme_precip_lead6	placebo	1.065	(0.933, 1.215)	0.351
D. Placebo tests	Coliform ~ extreme_precip	reference	1.142	(1.057, 1.235)	< 0.001
D. Placebo tests	Turbidity ~ smoke_10d_lead6	placebo	0.975	(0.930, 1.023)	0.308
E. Small-system sensitivity	E. coli	smoke_10d	1.084	(0.992, 1.185)	0.075
E. Small-system sensitivity	E. coli	tmax_mean	1.023	(1.004, 1.042)	0.016
E. Small-system sensitivity	Turbidity	extreme_precip	1.084	(1.046, 1.122)	< 0.001
E. Small-system sensitivity	E. coli	smoke_10d	1.098	(1.013, 1.189)	0.023
E. Small-system sensitivity	E. coli	tmax_mean	1.026	(1.008, 1.044)	0.004
E. Small-system sensitivity	Turbidity	extreme_precip	1.083	(1.047, 1.121)	< 0.001
E. Small-system sensitivity	Coliform	smoke_10d	1.084	(0.992, 1.185)	0.076
E. Small-system sensitivity	Coliform	extreme_precip	1.167	(1.078, 1.264)	< 0.001
E. Small-system sensitivity	DBP	extreme_precip	1.030	(1.006, 1.056)	0.016
F. Wild cluster bootstrap	Smoke -> E. coli	smoke_10d	1.098	(1.013, 1.189) [wild: (1.016, 1.186)]	0.023 [wild: 0.013]
F. Wild cluster bootstrap	Heat -> E. coli	tmax_mean	1.026	(1.008, 1.044) [wild: (1.008, 1.044)]	0.004 [wild: 0.001]
F. Wild cluster bootstrap	Precip -> Turbidity	extreme_precip	1.083	(1.047, 1.121) [wild: (1.047, 1.120)]	0.000 [wild: 0.001]
G. Smoke seasonality definitions	E. coli	smoke_10d	1.098	(1.013, 1.189)	0.023
G. Smoke seasonality definitions	E. coli	smoke_extreme_moy	1.025	(0.897, 1.170)	0.718
G. Smoke seasonality definitions	E. coli	smoke_z_hi	0.974	(0.883, 1.074)	0.595
G. Smoke seasonality definitions	E. coli	smoke_z	1.004	(0.971, 1.039)	0.808
G. Smoke seasonality definitions	E. coli	smoke_extreme_moy	1.057	(0.960, 1.164)	0.259

**Table:** 

Panel	Pair	Outcome	Specification	Estimate	95% CI	P value
A. Any-violation interaction summary	Wildfire smoke × extreme heat	Any violation	Both months = 2.16%	RERI - 0.092	(−0.135, −0.049)	< 0.001
A. Any-violation interaction summary	Wildfire smoke × extreme precipitation	Any violation	Both months = 1.09%	RERI - 0.016	(−0.071, 0.039)	0.573
A. Any-violation interaction summary	Extreme precipitation × extreme heat	Any violation	Both months = 0.22%	RERI - 0.040	(−0.114, 0.033)	0.285
B. Co-occurring-month estimates by outcome	Wildfire smoke × extreme heat	Any violation	Both	0.993	(0.961, 1.027)	0.692
B. Co-occurring-month estimates by outcome	Wildfire smoke × extreme heat	Turbidity	Both	1.083	(1.027, 1.141)	0.003
B. Co-occurring-month estimates by outcome	Wildfire smoke × extreme heat	Coliform	Both	1.084	(0.931, 1.262)	0.300
B. Co-occurring-month estimates by outcome	Wildfire smoke × extreme heat	DBP	Both	0.927	(0.857, 1.004)	0.064
B. Co-occurring-month estimates by outcome	Wildfire smoke × extreme heat	Treatment technique	Both	0.859	(0.762, 0.969)	0.014
B. Co-occurring-month estimates by outcome	Wildfire smoke × extreme heat	Monitoring	Both	0.944	(0.909, 0.980)	0.003
B. Co-occurring-month estimates by outcome	Wildfire smoke × extreme precipitation	Any violation	Both	1.088	(1.036, 1.142)	< 0.001
B. Co-occurring-month estimates by outcome	Wildfire smoke × extreme precipitation	Turbidity	Both	1.183	(1.092, 1.282)	< 0.001
B. Co-occurring-month estimates by outcome	Wildfire smoke × extreme precipitation	Coliform	Both	0.962	(0.764, 1.211)	0.739
B. Co-occurring-month estimates by outcome	Wildfire smoke × extreme precipitation	DBP	Both	1.097	(0.991, 1.214)	0.073
B. Co-occurring-month estimates by outcome	Wildfire smoke × extreme precipitation	Treatment technique	Both	1.001	(0.870, 1.151)	0.994
B. Co-occurring-month estimates by outcome	Wildfire smoke × extreme precipitation	Monitoring	Both	1.126	(1.056, 1.200)	< 0.001
B. Co-occurring-month estimates by outcome	Extreme precipitation × extreme heat	Any violation	Both	1.056	(0.985, 1.131)	0.124
B. Co-occurring-month estimates by outcome	Extreme precipitation × extreme heat	Turbidity	Both	1.325	(1.171, 1.498)	< 0.001
B. Co-occurring-month estimates by outcome	Extreme precipitation × extreme heat	Coliform	Both	1.308	(0.758, 2.259)	0.335
B. Co-occurring-month estimates by outcome	Extreme precipitation × extreme heat	DBP	Both	1.163	(0.975, 1.387)	0.093
B. Co-occurring-month estimates by outcome	Extreme precipitation × extreme heat	Treatment technique	Both	1.033	(0.784, 1.360)	0.819
B. Co-occurring-month estimates by outcome	Extreme precipitation × extreme heat	Monitoring	Both	1.093	(1.004, 1.191)	0.041

**Table:** 

Hazard	Outcome	Specification	RR	95% CI	P value	N
Extreme heat	E. coli	Baseline (PWS + ym)	1.026	(1.008, 1.045)	0.005	697,771
Extreme heat	E. coli	+ Month-of-year	1.030	(1.016, 1.045)	< 0.001	720,876
Extreme heat	E. coli	+ Region × month	1.029	(1.011, 1.047)	0.001	720,876
Extreme heat	E. coli	+ State × month	1.028	(1.011, 1.045)	0.001	713,206
Extreme heat	E. coli	+ County × month	1.027	(1.009, 1.046)	0.003	376,389
Extreme heat	E. coli	+ County × month + weather	1.031	(1.013, 1.050)	< 0.001	376,389
Extreme precipitation	DBP	Baseline (PWS + ym)	1.132	(1.067, 1.200)	< 0.001	2,369,970
Extreme precipitation	DBP	+ Month-of-year	1.131	(1.072, 1.193)	< 0.001	2,509,380
Extreme precipitation	DBP	+ Region × month	1.091	(1.028, 1.158)	0.004	2,509,380
Extreme precipitation	DBP	+ State × month	1.088	(1.026, 1.153)	0.005	2,058,255
Extreme precipitation	DBP	+ County × month	1.091	(1.030, 1.155)	0.003	1,077,960
Extreme precipitation	DBP	+ County × month + weather	1.100	(1.035, 1.169)	0.002	1,077,960
Extreme precipitation	E. coli	Baseline (PWS + ym)	1.116	(1.035, 1.203)	0.004	697,771
Extreme precipitation	E. coli	+ Month-of-year	1.093	(1.021, 1.171)	0.011	720,876
Extreme precipitation	E. coli	+ Region × month	1.105	(1.027, 1.189)	0.008	720,876
Extreme precipitation	E. coli	+ State × month	1.093	(1.002, 1.193)	0.046	713,206
Extreme precipitation	E. coli	+ County × month	1.085	(0.993, 1.186)	0.071	376,389
Extreme precipitation	E. coli	+ County × month + weather	1.117	(1.021, 1.223)	0.016	376,389
Extreme precipitation	Treatment technique	Baseline (PWS + ym)	1.100	(1.043, 1.160)	< 0.001	1,221,480
Extreme precipitation	Treatment technique	+ Month-of-year	1.110	(1.054, 1.169)	< 0.001	1,221,480
Extreme precipitation	Treatment technique	+ Region × month	1.122	(1.061, 1.187)	< 0.001	1,221,480
Extreme precipitation	Treatment technique	+ State × month	1.096	(1.034, 1.162)	0.002	1,217,985
Extreme precipitation	Treatment technique	+ County × month	1.105	(1.039, 1.175)	0.001	712,935
Extreme precipitation	Treatment technique	+ County × month + weather	1.107	(1.038, 1.181)	0.002	712,935
Extreme precipitation	Turbidity	Baseline (PWS + ym)	1.086	(1.045, 1.128)	< 0.001	3,540,915
Extreme precipitation	Turbidity	+ Month-of-year	1.089	(1.047, 1.134)	< 0.001	4,406,472
Extreme precipitation	Turbidity	+ Region × month	1.082	(1.039, 1.126)	< 0.001	4,406,472
Extreme precipitation	Turbidity	+ State × month	1.089	(1.051, 1.129)	< 0.001	4,406,430
Extreme precipitation	Turbidity	+ County × month	1.089	(1.048, 1.132)	< 0.001	3,758,090
Extreme precipitation	Turbidity	+ County × month + weather	1.084	(1.043, 1.126)	< 0.001	3,758,090
Wildfire smoke	E. coli	Baseline (PWS + ym)	1.278	(1.096, 1.490)	0.002	611,785
Wildfire smoke	E. coli	+ Month-of-year	1.269	(1.110, 1.449)	< 0.001	633,582
Wildfire smoke	E. coli	+ Region × month	1.213	(1.058, 1.390)	0.006	633,582
Wildfire smoke	E. coli	+ State × month	1.117	(0.995, 1.255)	0.062	625,254
Wildfire smoke	E. coli	+ County × month	1.116	(0.977, 1.273)	0.105	313,725
Wildfire smoke	E. coli	+ County × month + weather	1.188	(1.055, 1.337)	0.004	293,380

**Table:** 

Outcome	Exposure	Coefficient	SE	P value	N	Counties
log(turbidity_median)	extreme_precip (top decile)	0.502	0.066	< 0.001	92,620	1,141
log(turbidity_median)	precipitation_z	0.234	0.023	< 0.001	92,620	1,141
log(turbidity_median)	smoke_treat_10d	−0.097	0.036	0.018	92,620	1,141
log(turbidity_median)	hot_month_90	−0.181	0.026	< 0.001	92,620	1,141
log(turbidity_median)	heat_z	−0.379	0.062	< 0.001	92,620	1,141
log(turbidity_median)	extreme_precip (combined)	0.491	0.065	< 0.001	92,620	1,141
log(turbidity_median)	smoke_treat_10d (combined)	−0.052	0.032	0.135	92,620	1,141
log(turbidity_median)	hot_month_90 (combined)	−0.113	0.023	< 0.001	92,620	1,141
log(turbidity_p90)	extreme_precip (top decile)	0.582	0.079	< 0.001	92,620	1,141
log(turbidity_p90)	hot_month_90	−0.187	0.036	< 0.001	92,620	1,141
log(ecoli_median)	extreme_precip (top decile)	0.434	0.067	< 0.001	48,900	944
log(ecoli_median)	hot_month_90	−0.128	0.054	0.033	48,900	944
log(ecoli_median)	smoke_treat_10d	0.016	0.037	0.666	48,900	944
turbidity_median	n_violations (Spearman)	−0.029	NA	< 0.001	92,660	1,141

**Table:** 

Panel	Analysis	Specification	Estimate	95% CI	P value	N	Term	RR
A. Outcome-specific heat DLNM	E. coli	Lag 0 month	RR 1.494	(1.300, 1.718)				
A. Outcome-specific heat DLNM	E. coli	Lag 1 month	RR 1.315	(1.202, 1.440)				
A. Outcome-specific heat DLNM	E. coli	Lag 2 month	RR 1.175	(1.073, 1.287)				
A. Outcome-specific heat DLNM	E. coli	Lag 3 month	RR 1.082	(0.979, 1.196)				
A. Outcome-specific heat DLNM	E. coli	Lag 4 month	RR 1.037	(0.948, 1.134)				
A. Outcome-specific heat DLNM	E. coli	Lag 5 month	RR 1.024	(0.921, 1.137)				
A. Outcome-specific heat DLNM	E. coli	Lag 6 month	RR 1.026	(0.861, 1.221)				
A. Outcome-specific heat DLNM	Coliform	Lag 0 month	RR 1.494	(1.299, 1.717)				
A. Outcome-specific heat DLNM	Coliform	Lag 1 month	RR 1.315	(1.201, 1.439)				
A. Outcome-specific heat DLNM	Coliform	Lag 2 month	RR 1.175	(1.073, 1.287)				
A. Outcome-specific heat DLNM	Coliform	Lag 3 month	RR 1.082	(0.979, 1.197)				
A. Outcome-specific heat DLNM	Coliform	Lag 4 month	RR 1.038	(0.949, 1.135)				
A. Outcome-specific heat DLNM	Coliform	Lag 5 month	RR 1.025	(0.923, 1.139)				
A. Outcome-specific heat DLNM	Coliform	Lag 6 month	RR 1.028	(0.864, 1.224)				
B. Population weighting	Smoke × E. coli	Unweighted	RR 1.098	(1.013, 1.189)	0.023	676,861		
B. Population weighting	Smoke × E. coli	Population-weighted	RR 1.222	(1.001, 1.492)	0.049	676,861		
B. Population weighting	Heat × E. coli	Unweighted	RR 1.026	(1.008, 1.044)	0.004	676,861		
B. Population weighting	Heat × E. coli	Population-weighted	RR 1.071	(1.048, 1.093)	< 0.001	676,861		
B. Population weighting	Precip × Turbidity	Unweighted	RR 1.083	(1.047, 1.121)	< 0.001	3,306,893		
B. Population weighting	Precip × Turbidity	Population-weighted	RR 1.053	(0.972, 1.141)	0.204	3,306,893		
C. Excluding 2020	Smoke × E. coli	2006–2019	RR 1.102	(1.008, 1.205)	0.032	592,363		
C. Excluding 2020	Smoke × E. coli	2006–2020	RR 1.098	(1.013, 1.189)	0.023	676,861		
C. Excluding 2020	Heat × E. coli	2006–2019	RR 1.024	(1.009, 1.039)	0.002	592,363		
C. Excluding 2020	Heat × E. coli	2006–2020	RR 1.026	(1.008, 1.044)	0.004	676,861		
C. Excluding 2020	Precip × Turbidity	2006–2019	RR 1.083	(1.047, 1.121)	< 0.001	3,306,893		
C. Excluding 2020	Precip × Turbidity	2006–2020	RR 1.083	(1.047, 1.121)	< 0.001	3,306,893		
C. Excluding 2020	Precip × Coliform	2006–2019	RR 1.156	(1.072, 1.247)	< 0.001	593,336		
C. Excluding 2020	Precip × Coliform	2006–2020	RR 1.136	(1.057, 1.222)	< 0.001	678,220		
D. Goldilocks cutpoints	Smoke × E. coli	200–1000 (primary)	RR 1.234	(1.109, 1.373)	< 0.001	555,012		
D. Goldilocks cutpoints	Smoke × E. coli	200–1000 (primary) + county×MOY + weather	RR 1.182	(1.049, 1.333)	0.006	287,278		
D. Goldilocks cutpoints	Smoke × E. coli	150–800	RR 1.259	(1.152, 1.377)	< 0.001	561,814		
D. Goldilocks cutpoints	Smoke × E. coli	150–800 + county×MOY + weather	RR 1.172	(1.061, 1.295)	0.002	292,739		
D. Goldilocks cutpoints	Smoke × E. coli	150–1200	RR 1.238	(1.122, 1.366)	< 0.001	576,317		
D. Goldilocks cutpoints	Smoke × E. coli	150–1200 + county×MOY + weather	RR 1.172	(1.054, 1.303)	0.003	299,024		
D. Goldilocks cutpoints	Smoke × E. coli	300–800	RR 1.279	(1.119, 1.462)	< 0.001	529,285		
D. Goldilocks cutpoints	Smoke × E. coli	300–800 + county×MOY + weather	RR 1.217	(1.047, 1.414)	0.010	273,193		
D. Goldilocks cutpoints	Smoke × E. coli	300–1200	RR 1.238	(1.075, 1.427)	0.003	543,582		
D. Goldilocks cutpoints	Smoke × E. coli	300–1200 + county×MOY + weather	RR 1.211	(1.041, 1.409)	0.013	279,428		
E. Smoke threshold (coliform)	Coliform ~ >=5d smoke days	PWS + year-month FE		(1.027, 1.177)	0.006		smoke_5d	1.099
E. Smoke threshold (coliform)	Coliform ~ >=10d smoke days	PWS + year-month FE		(1.024, 1.222)	0.013		smoke_10d	1.119
E. Smoke threshold (coliform)	Coliform ~ >=15d smoke days	PWS + year-month FE		(1.033, 1.322)	0.013		smoke_15d	1.169
F. Precipitation threshold (turbidity)	Turbidity ~ P80	PWS + year-month FE		(1.035, 1.098)	< 0.001		extreme_p80	1.066
F. Precipitation threshold (turbidity)	Turbidity ~ P90	PWS + year-month FE		(1.045, 1.128)	< 0.001		extreme_p90	1.086
F. Precipitation threshold (turbidity)	Turbidity ~ P95	PWS + year-month FE		(1.071, 1.168)	< 0.001		extreme_p95	1.119

## Supplementary Material

1

## Figures and Tables

**Table 1 |:** Study sample characteristics.

***Water system characteristics***	
Community water systems, N	56,351
State/territory clusters	49
System-months observed	9.0 million
Median population served (IQR)	338 (90–1,800)
Surface water source, %	7.2
Small systems (<3,300 served), %	82.4
Publicly owned, %	48.7
***Exposure prevalence (county-level)***	
County-months observed	648,360
Wildfire smoke (≥10 days), ***%***	12.3
Hot county-months (>P95 of Tmax; descriptive), %	4.3
Extreme precipitation (>P90), %	8.6
Compound smoke × heat, %	2.2
***Violation rates (per 10,000 system-months)***	
Any SDWA violation	1390.3
Monitoring	1067.0
DBP	327.4
Health-based	310.6
Arsenic	195.7
Nitrate	195.2
SWTR	180.6
Turbidity	108.5
Lead/Copper	106.1
Treatment technique	28.8
TOC	15.8
Coliform	8.94
E. coli	8.93

**Table 2 |:** Hazard-specific evidence hierarchy, mechanism probes, and policy translation.

Hazard	Main compliance signature	Monthly targeting signal	Measured-water pattern	Mechanism probes	Pathway and policy translation
**Extreme precipitation**	Turbidity RR 1.083; broad screen positive	0.998 (P 0.798)	Turbidity +65%; E. coli +54%; treated-water turbidity +29.4%; Daily turbidity +52.8%; daily E. coli +68.2%	HUC8 turbidity RR 1.089 vs county RR 1.037; flowing-site RR 1.330; discharge attenuates turbidity by 54.6%; No parallel outage-based mechanism required; Inspection RR 0.998; enforcement RR 1.035	Primary source-water disruption with downstream compliance amplification; Best case for post-storm triggered follow-up, especially for turbidity/coliform
**Extreme heat**	E. coli RR 1.026 per °C	0.984 (P 0.142)	Turbidity −17%; E. coli −12%; treated-water turbidity −10.9% (NS); Daily turbidity +19.9%; daily E. coli +41.1%; warm-day burden E. coli +13.0%	Groundwater RR 1.029; surface RR 0.999; Hot months increase outage intensity (P <0.001); Inspection RR 1.009; enforcement RR 1.001	Operational/distribution stress more consistent than upstream source deterioration; Tentative hot-period microbial follow-up; weaker than precipitation
**Wildfire smoke**	Pooled E. coli RR 1.098; transport-zone month-matched enrichment 1.279	1.279 (P <0.001)	Turbidity −9%; E. coli +2%; treated-water turbidity +7.1% (NS); Daily turbidity −3.4%; daily E. coli +1.4%; 500–1000 km lag-4 E. coli +5.4%; HMS plume lag-4 E. coli +20.7%	Groundwater RR 1.089; surface interaction RR 0.783; No outage increase; transport estimate unchanged with outage control; Inspection RR 1.031; enforcement RR 1.005; transport enforcement RR 0.901	Identification-sensitive transport probe, not a resolved national source-water pathway; Exploratory transport-zone attention only; not a general smoke trigger

## Data Availability

All violation and Lead and Copper Rule customer-tap sample data are publicly available from the EPA SDWIS database (https://www.epa.gov/ground-water-and-drinking-water/safe-drinking-water-information-system-sdwis-federal-reporting). Wildfire smoke PM_2.5_ estimates are from Childs et al. (available at https://github.com/echolab-stanford/daily-10km-smokePM). PRISM temperature and precipitation data are available from the PRISM Climate Group (https://prism.oregonstate.edu). MTBS fire perimeters are available from the USGS (https://www.mtbs.gov). Water quality measurements are from the EPA/USGS Water Quality Portal (https://www.waterqualitydata.us). USGS stream discharge data are from the National Water Information System (https://waterservices.usgs.gov). EPA community water system service-area boundaries are available at https://www.epa.gov/ground-water-and-drinking-water/public-water-system-service-areas. NOAA Hazard Mapping System smoke polygons are available at https://satepsanone.nesdis.noaa.gov/pub/FIRE/web/HMS/Smoke_Polygons/. EAGLE-I power outage data are available via Figshare (https://doi.org/10.6084/m9.figshare.24237376.v4).

## Code availability

Full analysis code, manuscript source files, render scripts, and key intermediate model-output tables sufficient to reproduce the submitted figures and tables are provided to editors and reviewers in a confidential review package at submission and will be deposited in a public repository upon publication.
