# Supplementary material for: Climate hazards expose distinct compliance vulnerabilities in US drinking water systems
Source: Res Sq. 2026 Apr 15:rs.3.rs-9217282. Preprint. [Version 1] doi: 10.21203/rs.3.rs-9217282/v1 (PMC13105104; doi:10.21203/rs.3.rs-9217282/v1)
Supplement: 1 [file NIHPPRS9217282V1-supplement-1.pdf]

Supplementary Information

Supplementary Figure 1 | Robustness checks. a, Wildfire smoke–turbidity rate ratios across alternative specifications: threshold sensitivity (5, 10, 15 smoke days), exclusion of extreme fire years, and continuous smoke-day dose. *Continuous smoke days is a per-day effect with smaller magnitude by construction. **b**, Burn scar × precipitation interaction across five sensitivity specifications. All burn scar specifications are non-significant.

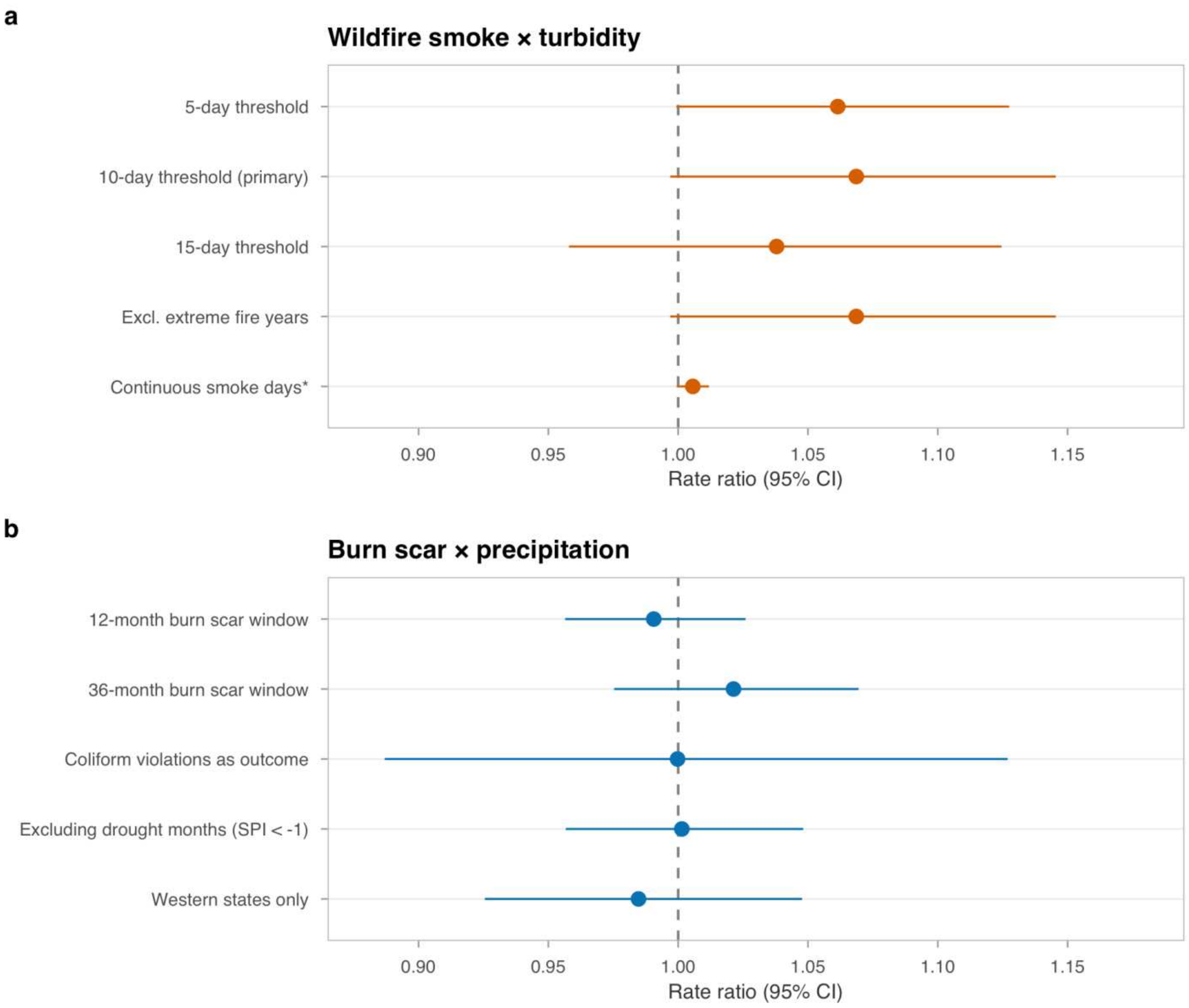

Supplementary Figure 2 | Event study for wildfire smoke and turbidity violations. Pre-treatment trends and post-exposure dynamics for turbidity violation counts, showing violation rates in months before and after wildfire smoke events (≥10 smoke days). County and year-month fixed effects with state-clustered standard errors. Pre-treatment coefficients are generally centred near zero, though noisy; the data do not reject the parallel trends assumption, but limited pre-event precision means that modest violations cannot be ruled out. Event studies for other hazard–outcome pairs (smoke–*E. coli*, precipitation–turbidity) are not shown; similar pre-trend tests would strengthen the parallel trends evidence for the primary findings.

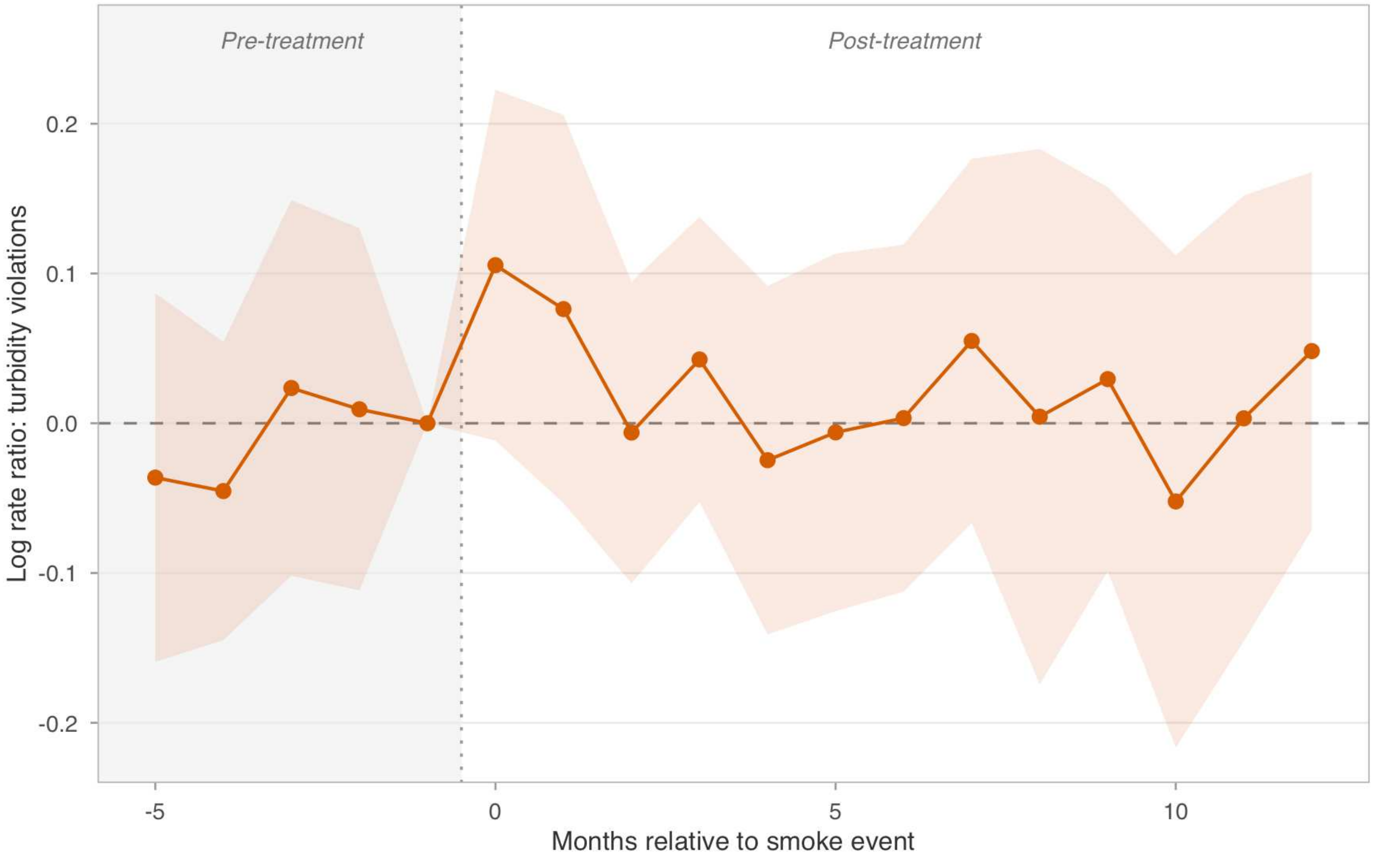

Supplementary Figure 3 | Distributed lag non-linear model for extreme heat effects on composite violations. **a**, Cumulative relative risk of any SDWA violation as a function of monthly maximum temperature, referenced to the county-specific median. Grey shading marks the 5th and 95th percentile tails where data is sparse and estimates reflect extrapolation. **b**, Lag-specific effects at the 95th percentile of temperature, showing a delayed heat-risk window: relative risk is elevated at lags 3–3 months and peaks at lag 3 (RR = 1.026; 95% CI: 1.003–1.050). This composite any-violation DLNM aggregates across twelve violation categories, most of which are unresponsive to heat; the per-°C *E. coli* result (main text) isolates the primary microbial signal. Pink shaded regions indicate 95% confidence intervals.

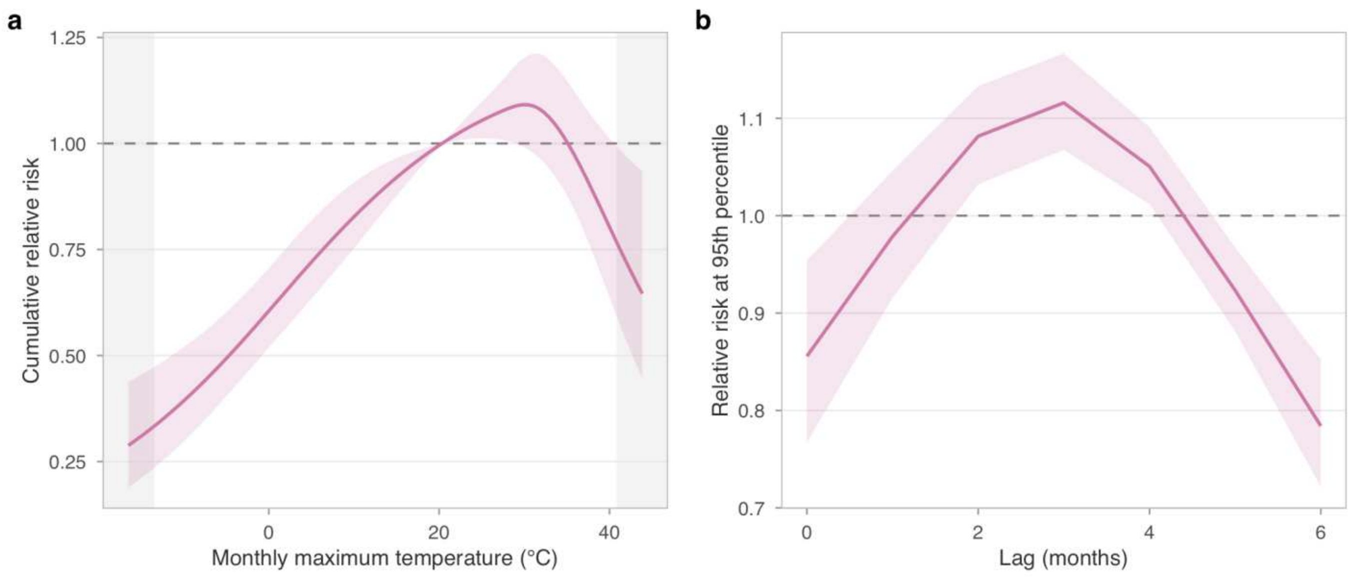

Division	EJ variable	Interaction RR	95% CI	P value	q value	N
National	pct_minority	1.0160	0.977–1.057	0.4302	0.646	9,021,476
National	pct_poverty	0.9502	0.814–1.109	0.5169	0.646	9,021,476
E North Central	pct_minority	0.9246	0.775–1.104	0.3852	0.646	1,118,270
E North Central	pct_poverty	0.6261	0.303–1.295	0.2068	0.517	1,118,270
E South Central	pct_minority	0.9665	0.732–1.276	0.8099	0.852	462,266
E South Central	pct_poverty	0.3808	0.229–0.632	< 0.001	0.002	462,266
Mid Atlantic	pct_minority	1.2524	1.131–1.386	< 0.001	< 0.001	912,340
Mid Atlantic	pct_poverty	0.6294	0.170–2.333	0.4886	0.646	912,340
Mountain	pct_minority	0.9743	0.913–1.039	0.4306	0.646	915,230
Mountain	pct_poverty	0.9365	0.732–1.199	0.6023	0.669	915,230
New England	pct_minority	1.1982	1.015–1.414	0.0322	0.129	475,342
New England	pct_poverty	0.6947	0.438–1.103	0.1222	0.349	475,342
Pacific	pct_minority	0.9485	0.893–1.008	0.0869	0.290	1,189,653
Pacific	pct_poverty	0.9247	0.787–1.087	0.3420	0.646	1,189,653
South Atlantic	pct_minority	1.1013	1.037–1.169	0.0016	0.008	1,541,514
South Atlantic	pct_poverty	0.9473	0.824–1.089	0.4481	0.646	1,541,514
W North Central	pct_minority	1.0022	0.829–1.212	0.9820	0.982	1,080,130
W North Central	pct_poverty	0.8825	0.575–1.355	0.5677	0.668	1,080,130
W South Central	pct_minority	1.0407	1.019–1.063	< 0.001	0.002	1,326,731
W South Central	pct_poverty	1.1440	0.806–1.624	0.4519	0.646	1,326,731

Supplementary Table 2 | Independent WQP sampling intensity during smoke months by fire-distance bin. County-month counts of sampled WQP sites and sampled site-days, aggregated from daily WQP panels for turbidity and E. coli, regressed on smoke distance bins relative to no-smoke months. Models use county and year-month fixed effects with state-clustered standard errors. If local-fire emergencies suppressed independent monitoring strongly enough to explain the transported-smoke result, sampling intensity would be expected to fall across transported-smoke bins; instead, declines are limited to the local 0–100 km E. coli site-count specification and transported-smoke bins are generally null or positive.

Outcome	Metric	Distance to nearest fire	RR	95% CI	P value
Daily turbidity	Sampled sites	0-100 km	0.956	0.836–1.094	0.514
Daily turbidity	Sampled sites	100-200 km	1.000	0.880–1.137	0.999
Daily turbidity	Sampled sites	1000+ km	1.203	0.950–1.524	0.125
Daily turbidity	Sampled sites	200-500 km	1.131	1.063–1.202	< 0.001
Daily turbidity	Sampled sites	500-1000 km	1.105	0.961–1.269	0.161
Daily turbidity	Sampled site-days	0-100 km	0.946	0.821–1.089	0.438
Daily turbidity	Sampled site-days	100-200 km	0.961	0.830–1.114	0.600
Daily turbidity	Sampled site-days	1000+ km	1.440	0.974–2.130	0.068
Daily turbidity	Sampled site-days	200-500 km	1.180	1.080–1.290	< 0.001
Daily turbidity	Sampled site-days	500-1000 km	1.214	0.902–1.633	0.201
Daily E. coli	Sampled sites	0-100 km	0.902	0.802–1.014	0.083
Daily E. coli	Sampled sites	100-200 km	0.971	0.803–1.175	0.765
Daily E. coli	Sampled sites	1000+ km	2.066	1.419–3.009	< 0.001
Daily E. coli	Sampled sites	200-500 km	0.993	0.835–1.181	0.939
Daily E. coli	Sampled sites	500-1000 km	1.746	1.526–1.998	< 0.001
Daily E. coli	Sampled site-days	0-100 km	0.875	0.719–1.065	0.184
Daily E. coli	Sampled site-days	100-200 km	0.947	0.716–1.254	0.706
Daily E. coli	Sampled site-days	1000+ km	2.225	1.519–3.259	< 0.001
Daily E. coli	Sampled site-days	200-500 km	1.141	0.908–1.435	0.256

Outcome	Metric	Distance to nearest fire	RR	95% CI	P value
Daily E. coli	Sampled site-days	500-1000 km	1.948	1.635–2.321	< 0.001

Supplementary Table 3 | Source-architecture stratification across main hazard pathways. Panel A reports source-type-specific rate ratios for the main hazard-outcome pairs discussed in the text. Panel B reports direct surface-versus-groundwater interaction terms. The heat-*E. coli* signal is concentrated in groundwater systems rather than surface systems, which is more consistent with operational or distribution-system stress than with broad surface-source-water deterioration. All models use PWS and year-month fixed effects with state-clustered standard errors.

Panel	Hazard	Outcome	Category	RR	95% CI	P value
A. Main source-type strata	Wildfire smoke	E. coli	Surface	1.045	0.797–1.371	0.749
A. Main source-type strata	Wildfire smoke	E. coli	Groundwater	1.089	0.993–1.195	0.069
A. Main source-type strata	Extreme heat	E. coli	Surface	0.999	0.955–1.045	0.960
A. Main source-type strata	Extreme heat	E. coli	Groundwater	1.029	1.009–1.049	0.004
A. Main source-type strata	Extreme precipitation	Any violation	Surface	1.040	1.017–1.063	< 0.001
A. Main source-type strata	Extreme precipitation	Any violation	Groundwater	1.032	1.014–1.049	< 0.001
A. Main source-type strata	Extreme precipitation	Turbidity	Surface	1.060	0.945–1.188	0.320
A. Main source-type strata	Extreme precipitation	Turbidity	Groundwater	1.087	1.049–1.127	< 0.001
B. Surface-versus-groundwater interaction	Wildfire smoke	E. coli	smoke_10d:is_surface	0.783	0.605–1.013	0.063
B. Surface-versus-groundwater interaction	Extreme heat	E. coli	tmax_mean:is_surface	0.973	0.963–0.982	< 0.001

Panel	Hazard	Outcome	Category	RR	95% CI	P value
B. Surface-versus-groundwater interaction	Extreme precipitation	Any violation	extreme_precip:is_surface	1.014	0.988–1.042	0.286
B. Surface-versus-groundwater interaction	Extreme precipitation	Turbidity	extreme_precip:is_surface	0.991	0.887–1.106	0.866
B. Surface-versus-groundwater interaction	Extreme precipitation	Coliform	extreme_precip:is_surface	0.983	0.751–1.287	0.901
B. Surface-versus-groundwater interaction	Extreme precipitation	DBP	extreme_precip:is_surface	1.011	0.960–1.064	0.689

Supplementary Table 4 | Service-area reassignment sensitivity using EPA public water system boundaries. Headline hazard-outcome models are re-estimated in the subset of systems with public EPA community water system polygons, comparing the default county-of-record assignment with both the representative-point proxy and true area-weighted county overlap. Matched systems represent 43,699 systems (48.0% of the analytic sample) and 83.7% of served population; mean county overlap is 1.21 counties per matched system.

Hazard	Outcome	Exposure assignment	RR	95% CI	P value	N
Wildfire smoke	E. coli	County of record	1.101	1.006–1.206	0.036	595,544
Wildfire smoke	E. coli	Service-area point	1.104	1.011–1.206	0.028	595,544
Wildfire smoke	E. coli	Service-area area-weighted	1.105	1.008–1.211	0.033	595,544
Extreme heat	E. coli	County of record	1.022	1.004–1.041	0.019	584,521
Extreme heat	E. coli	Service-area point	1.025	1.009–1.041	0.002	595,544
Extreme heat	E. coli	Service-area area-weighted	1.025	1.009–1.041	0.002	595,544
Extreme precipitation	Turbidity	County of record	1.094	1.051–1.139	< 0.001	2,701,530
Extreme precipitation	Turbidity	Service-area point	1.096	1.053–1.141	< 0.001	2,728,180
Extreme precipitation	Turbidity	Service-area area-weighted	1.098	1.055–1.143	< 0.001	2,728,180

Supplementary Table 5 | Alternative smoke exposure sensitivity using NOAA HMS polygons. Smoke-*E. coli* models are re-estimated using an alternative county-month smoke-day panel built from NOAA Hazard Mapping System daily smoke polygons and county representative points. The county-month smoke-day correlation between the HMS and Childs exposure definitions is 0.92. The Goldilocks comparison uses the same 200–1,000 km active-fire restriction and concurrent weather controls.

Exposure definition	Specification	RR	95% CI	P value	N
Childs smoke PM2.5	Pooled baseline	1.098	1.013–1.189	0.023	676,861
NOAA HMS polygons	Pooled baseline	1.131	1.000–1.279	0.051	676,861
Childs smoke PM2.5	Goldilocks + weather	1.157	1.027–1.303	0.017	561,326
NOAA HMS polygons	Goldilocks + weather	1.134	0.952–1.351	0.159	561,326

Supplementary Table 6 | Daily USGS discharge mechanism probe in the WQP validation subset. USGS National Water Information System discharge is linked to the subset of WQP drinking-water monitoring sites with USGS station identifiers. Panel rows show fixed-effects estimates for discharge on weather/smoke and for turbidity or E. coli on the same covariates with and without discharge adjustment. Strong precipitation-discharge coupling and attenuation of the precipitation coefficients after discharge adjustment are consistent with a source-water transport pathway.

Outcome	Specification	Term	Coefficient	SE	P value	Attenuation (%)	N observations	N sites
Daily turbidity	Discharge on weather	log(1 + precipitation)	0.377	0.027	< 0.001		16,240	321
Daily turbidity	Discharge on weather	Maximum temperature	-0.006	0.005	0.203		16,240	321
Daily turbidity	Discharge on weather	log(1 + smoke PM2.5)	-0.027	0.027	0.318		16,240	321
Daily turbidity	Turbidity baseline	log(1 + precipitation)	0.503	0.025	< 0.001	54.6	16,240	321
Daily turbidity	Turbidity baseline	Maximum temperature	0.007	0.008	0.372	-63.1	16,240	321
Daily turbidity	Turbidity baseline	log(1 + smoke PM2.5)	-0.062	0.030	0.039		16,240	321
Daily turbidity	Turbidity + discharge	log(1 + precipitation)	0.229	0.027	< 0.001	54.6	16,240	321
Daily turbidity	Turbidity + discharge	Maximum temperature	0.011	0.005	0.026	-63.1	16,240	321
Daily turbidity	Turbidity + discharge	log(1 + smoke PM2.5)	-0.043	0.018	0.017		16,240	321
Daily turbidity	Turbidity + discharge	log(discharge)	0.728	0.053	< 0.001		16,240	321

Outcome	Specification	Term	Coefficient	SE	P value	Attenuation (%)	N observations	N sites
Daily E. coli	E. coli baseline	log(1 + precipitation)	0.638	0.032	< 0.001	34.0	10,227	134
Daily E. coli	E. coli baseline	Maximum temperature	-0.018	0.007	0.013		10,227	134
Daily E. coli	E. coli baseline	log(1 + smoke PM2.5)	-0.075	0.037	0.049		10,227	134
Daily E. coli	E. coli + discharge	log(1 + precipitation)	0.421	0.047	< 0.001	34.0	10,227	134
Daily E. coli	E. coli + discharge	Maximum temperature	-0.008	0.006	0.182		10,227	134
Daily E. coli	E. coli + discharge	log(1 + smoke PM2.5)	-0.056	0.018	0.003		10,227	134
Daily E. coli	E. coli + discharge	log(discharge)	0.645	0.117	< 0.001		10,227	134

Supplementary Table 7 | EAGLE-I county-month outage mechanism probe. County-level EAGLE-I outage metrics are aggregated to county-months for 2014-2020. The first rows test whether heat or smoke months predict outage occurrence or outage intensity under county and year-month fixed effects. The remaining rows add county-month outage intensity to the heat-E. coli and transport-smoke E. coli PWS models as a coarse operational mediation control. Hot months increase outage intensity, but smoke months do not, and outage adjustment leaves the PWS coefficients essentially unchanged.

Outcome	Specification	Term	Estimate	95% CI	P value	N
Any county outage month	Heat -> outage	Hot month (top decile)	RR 1.001	0.974-1.029	0.941	222,444
Log monthly max outage share	Heat -> outage	Hot month (top decile)	b = 0.087	0.043-0.132	< 0.001	258,720
Any county outage month	Smoke pooled -> outage	Smoke month (>=10 d)	RR 1.006	0.983-1.030	0.607	222,444
Log monthly max outage share	Smoke pooled -> outage	Smoke month (>=10 d)	b = 0.006	-0.045-0.056	0.829	258,720
Any county outage month	Smoke transport -> outage	Transport smoke (200-1,000 km)	RR 1.005	0.978-1.032	0.734	222,444
Log monthly max outage share	Smoke transport -> outage	Transport smoke (200-1,000 km)	b = 0.002	-0.046-0.051	0.923	258,720
E. coli violations	Heat baseline	Mean Tmax	RR 1.024	1.004-1.044	0.017	260,064
E. coli violations	Heat + outage control	Mean Tmax	RR 1.024	1.004-1.044	0.017	260,064
E. coli violations	Heat + outage control	Monthly max outage share	RR 1.018	0.920-1.127	0.723	260,064
E. coli violations	Smoke transport baseline	Transport smoke (200-1,000 km)	RR 1.182	1.045-1.336	0.008	215,700
E. coli violations	Smoke transport + outage control	Transport smoke (200-1,000 km)	RR 1.182	1.045-1.337	0.008	215,700

Outcome	Specification	Term	Estimate	95% CI	P value	N
E. coli violations	Smoke transport + outage control	Monthly max outage share	RR 1.012	0.919-1.115	0.811	215,700

Supplementary Table 8 | Source-water-focused daily WQP validation. Daily WQP precipitation models are summarized for flowing versus standing hydro-classes, which serve as a coarse proxy for source-water-like versus less source-water-like monitoring contexts. The stronger turbidity response in flowing sites supports a source-water runoff pathway for precipitation.

Outcome	Comparison	Hazard	Specification	Estimate	95% CI	P value	N observations
Daily turbidity	Flowing	Precipitation	Best lag	42.1%	37.9 to 46.4%	< 0.001	451,502
Daily turbidity	Standing	Precipitation	Best lag	3.0%	1.9 to 4.0%	< 0.001	71,937
Daily turbidity	Flowing vs standing interaction	Precipitation	Interaction	RR 1.330	1.259-1.406	< 0.001	523,439
Daily E. coli	Flowing	Precipitation	Best lag	55.1%	48.1 to 62.4%	< 0.001	186,702
Daily E. coli	Standing	Precipitation	Best lag	54.6%	39.7 to 71.2%	< 0.001	33,191
Daily E. coli	Flowing vs standing interaction	Precipitation	Interaction	RR 1.097	0.996-1.208	0.061	219,893

Supplementary Table 9 | Within-site daily WQP case-crossover validation package. Site-day WQP panels are analyzed with site and year-month fixed effects, emphasizing same-day and mechanism-aligned windows. Precipitation and heat validate strongly in the within-site daily design, whereas smoke remains weak and lag-sensitive.

Outcome	Hazard	Alignment	Percent change	95% CI	P value	N observations	N sites	N counties
Daily E. coli	Precipitation	Same-day	68.2%	60.6 to 76.0%	< 0.001	109,332	6,527	526
Daily E. coli	Heat	Aligned heat window	41.1%	27.0 to 56.8%	< 0.001	109,332	6,527	526
Daily E. coli	Wildfire smoke	Same-day	-5.1%	-8.6 to -1.6%	0.005	109,332	6,527	526
Daily E. coli	Wildfire smoke	Aligned smoke lag	1.4%	-1.3 to 4.1%	0.314	109,332	6,527	526
Daily turbidity	Precipitation	Aligned precipitation window	52.8%	42.3 to 64.1%	< 0.001	203,566	7,898	785
Daily turbidity	Heat	Aligned heat window	19.9%	5.7 to 35.9%	0.005	203,566	7,898	785
Daily turbidity	Wildfire smoke	Same-day	-3.0%	-5.3 to -0.6%	0.016	203,566	7,898	785
Daily turbidity	Wildfire smoke	Aligned smoke window	-3.4%	-6.8 to -0.0%	0.050	203,566	7,898	785

Supplementary Table 10 | Counterfactual monitoring allocation analysis under equal-budget hazard-blind baselines. Observed hazard-trigger windows are compared with equal-budget blind timing within systems and within systems x calendar month. This is a coarse monthly counterfactual allocation exercise rather than a formal optimization model.

Hazard-outcome pair	Baseline	Triggered share	Observed capture share	Random capture share	Enrichment ratio	Trigger yield per 10k	Random yield per 10k	P value
Extreme precipitation -> Turbidity	Within-system random allocation	10.0%	10.3%	10.0%	1.034	61.09	59.07	< 0.001
Extreme precipitation -> Turbidity	Within-system x calendar-month random allocation	10.0%	10.3%	10.4%	0.998	61.09	61.23	0.798
Extreme heat -> E. coli	Within-system random allocation	23.0%	31.4%	23.2%	1.350	6.85	5.07	< 0.001
Extreme heat -> E. coli	Within-system x calendar-month random allocation	23.0%	31.4%	31.9%	0.984	6.85	6.96	0.142
Transport smoke -> E. coli	Within-system random allocation	6.2%	8.0%	4.9%	1.616	6.92	4.28	< 0.001
Transport smoke -> E. coli	Within-system x calendar-month random allocation	6.2%	8.0%	6.2%	1.279	6.92	5.41	< 0.0

Hazard-outcome pair	Baseline	Triggered share	Observed capture share	Random capture share	Enrichment ratio	Trigger yield per 10k	Random yield per 10k	P value
								01

Supplementary Table 11 | Exploratory daily WQP counterfactual monitoring allocation analysis for upper-tail turbidity days. Site-specific top-decile daily hazard triggers are compared with the upper tail of daily turbidity outcomes in the WQP site-day panels. Daily turbidity is shown because binary daily *E. coli* positivity is nearly universal in the available surface-site subset and is not informative for trigger prioritisation. The precipitation trigger captures a disproportionately large share of high-turbidity days relative to its sampling burden, whereas heat and smoke are much less concentrated.

Hazard	Trigger share	High-outcome share	High-outcome capture	Enrichment ratio	Triggered prevalence	Untriggered prevalence	N site-days
Precipitation	14.7%	13.2%	40.7%	2.77	0.365	0.092	574,120
Heat	12.8%	13.2%	19.0%	1.48	0.195	0.122	574,120
Smoke	38.5%	13.2%	43.8%	1.14	0.150	0.120	574,120

Supplementary Table 12 | Direct tap-side validation using national Lead and Copper Rule customer-tap samples. Customer-tap lead and copper samples are aggregated to monitoring-period 90th percentiles, indexed to the monitoring-period end month, and linked to same-month county climate exposures through the SDWIS county-of-record crosswalk. Models use PWS and year-month fixed effects with state-clustered standard errors. This is a coarser temporal validation than the WQP analyses because climate is aligned to the monitoring-period end month rather than the full monitoring window.

Contaminant	Outcome	Hazard	RR	95% CI	P value	N observations
Lead 90th percentile	Log monitoring-period 90th percentile	Wildfire smoke month (>=10 d)	0.963	0.892–1.040	0.345	231,040
Lead 90th percentile	Log monitoring-period 90th percentile	Maximum temperature (per °C)	1.004	0.997–1.012	0.276	231,040
Lead 90th percentile	Log monitoring-period 90th percentile	Extreme precipitation month	0.970	0.940–1.001	0.068	231,040
Lead 90th percentile	Monitoring-period action-level exceedance	Wildfire smoke month (>=10 d)	0.909	0.783–1.054	0.205	37,236
Lead 90th percentile	Monitoring-period action-level exceedance	Maximum temperature (per °C)	1.014	0.997–1.031	0.099	37,236
Lead 90th percentile	Monitoring-period action-level exceedance	Extreme precipitation month	0.973	0.900–1.052	0.489	37,236
Copper 90th percentile	Log monitoring-period 90th percentile	Wildfire smoke month (>=10 d)	0.962	0.926–0.999	0.051	5,022
Copper 90th percentile	Log monitoring-period 90th percentile	Maximum temperature (per °C)	1.003	0.999–1.006	0.129	5,022

Contaminant	Outcome	Hazard	RR	95% CI	P value	N observations
Copper 90th percentile	Log monitoring-period 90th percentile	Extreme precipitation month	1.016	0.993–1.039	0.189	5,022

Supplementary Table 13 | Hazard associations with SDWA site-visit activity. Public water system-month fixed-effects models estimate whether hazard months coincide with any site visit, sanitary surveys or assessments, enforcement or investigation visits, or visits documenting significant deficiencies in 0-1 month windows.

Hazard	Outcome	RR	95% CI	P value	N observations
Extreme precipitation	Any site visit (0-1 month window)	0.998	0.970-1.026	0.889	8,839,044
Extreme precipitation	Sanitary survey/assessment (0-1 month window)	0.992	0.961-1.023	0.601	8,828,958
Extreme precipitation	Enforcement/investigation visit (0-1 month window)	1.035	0.985-1.088	0.168	992,696
Extreme precipitation	Visit with significant deficiency (0-1 month window)	1.029	0.974-1.086	0.309	2,852,614
Extreme heat	Any site visit (0-1 month window)	1.009	1.000-1.018	0.063	8,839,044
Extreme heat	Sanitary survey/assessment (0-1 month window)	1.013	1.000-1.026	0.048	8,828,958
Extreme heat	Enforcement/investigation visit (0-1 month window)	1.001	0.993-1.010	0.768	992,696
Extreme heat	Visit with significant deficiency (0-1 month window)	1.008	0.994-1.022	0.265	2,852,614
Wildfire smoke	Any site visit (0-1 month window)	1.031	0.974-1.090	0.291	8,839,044
Wildfire smoke	Sanitary survey/assessment (0-1 month window)	1.013	0.951-1.079	0.696	8,828,958
Wildfire smoke	Enforcement/investigation visit (0-1 month window)	1.005	0.910-1.110	0.921	992,696
Wildfire smoke	Visit with significant deficiency (0-1 month window)	0.969	0.893-1.052	0.451	2,852,614
Transport smoke	Any site visit (0-1 month window)	1.018	0.965-1.075	0.507	8,839,044

Hazard	Outcome	RR	95% CI	P value	N observations
Transport smoke	Sanitary survey/assessment (0-1 month window)	1.005	0.941-1.073	0.884	8,828,958
Transport smoke	Enforcement/investigation visit (0-1 month window)	0.901	0.754-1.077	0.251	992,696
Transport smoke	Visit with significant deficiency (0-1 month window)	0.960	0.872-1.058	0.413	2,852,614

Supplementary Table 14 | HUC8 source-watershed reassignment sensitivity for precipitation. Surface-system precipitation models are re-estimated on the same HUC8-linked sample using county-of-record exposure assignment and a hydrologically aligned HUC8-month exposure assignment. Movement toward larger precipitation coefficients under HUC8 reassignment is consistent with attenuation from county-of-record intake misclassification.

Analysis	Exposure assignment	Outcome	Sample	RR	95% CI	P value	N observations
Precipitation -> turbidity	County-month	Turbidity	Surface systems with HUC8 assignment	1.037	0.932-1.154	0.503	173,647
Precipitation -> turbidity	HUC8-month	Turbidity	Surface systems with HUC8 assignment	1.089	0.996-1.190	0.061	173,647
Precipitation -> coliform	County-month	Coliform	Surface systems with HUC8 assignment	1.183	0.911-1.536	0.208	27,030
Precipitation -> coliform	HUC8-month	Coliform	Surface systems with HUC8 assignment	1.093	0.854-1.400	0.480	27,030

Supplementary Table 15 | Narrow treated-water-adjacent WQP turbidity validation. Monthly turbidity models are re-estimated in the subset of WQP monitoring locations tagged as public-water-supply facilities and closely related distribution/pipe locations. This subset is much smaller than the main WQP panel and is interpreted as a treated-water-adjacent probe rather than a definitive finished-water validation.

Specification	Exposure	Outcome	Percent change	95% CI	P value	N county-months	Counties	Sites
Strict PWS facility subset	Extreme precipitation	log(monthly median turbidity)	29.4%	23.6% to 35.5%	0.002	1,063	15	35
Strict PWS facility subset	Extreme heat	log(monthly median turbidity)	-10.9%	-36.6% to 25.1%	0.552	1,063	15	35
Strict PWS facility subset	Wildfire smoke	log(monthly median turbidity)	7.1%	-4.2% to 19.8%	0.316	1,063	15	35
Expanded treated/distribution subset	Extreme precipitation	log(monthly median turbidity)	20.2%	4.8% to 38.0%	0.058	1,122	28	71
Expanded treated/distribution subset	Extreme heat	log(monthly median turbidity)	-15.4%	-37.0% to 13.5%	0.327	1,122	28	71
Expanded treated/distribution subset	Wildfire smoke	log(monthly median turbidity)	-1.8%	-21.1% to 22.2%	0.878	1,122	28	71

Supplementary Table 16 | Daily WQP transport-smoke event study. Daily *E. coli* models are re-estimated with site × month fixed effects after restricting the sample to no-smoke months and smoke months in the 200–500 km and 500–1,000 km transport bands. Same-day and lag-4 smoke-intensity models are shown to distinguish local emergency timing from delayed far-transport timing.

Outcome	Sample	Alignment	Percent change per log-unit	95% CI	P value	N observations	Sites	Counties
Daily E. coli	Goldilocks 200-1000 km	Same-day smoke	-13.3%	-18.1% to -8.1%	< 0.001	32,022	2,995	383
Daily E. coli	Goldilocks 200-1000 km	Lag-4 smoke	-0.9%	-6.2% to 4.6%	0.739	32,022	2,995	383
Daily E. coli	Transport band 200-500 km	Same-day smoke	-13.7%	-19.3% to -7.8%	< 0.001	26,576	2,351	349
Daily E. coli	Transport band 200-500 km	Lag-4 smoke	-6.8%	-13.8% to 0.8%	0.081	26,576	2,351	349
Daily E. coli	Transport band 500-1000 km	Same-day smoke	-12.7%	-19.6% to -5.2%	0.001	27,595	2,601	350
Daily E. coli	Transport band 500-1000 km	Lag-4 smoke	5.4%	0.0% to 11.0%	0.050	27,595	2,601	350

Supplementary Table 17 | Daily WQP heat-persistence validation. Within-site × month models replace daily heat intensity with rolling counts of warm days over the prior 7 and 14 days, using site-specific P75-P90 daily temperature thresholds. The continuous 14-day mean-temperature model is shown for comparison.

Outcome	Metric	Threshold	Percent change over contrast	95% CI	P value	Contrast type	Exposure contrast	N observations
Daily E. coli	14-day mean temperature	Continuous	42.7%	27.8% to 59.2%	< 0.001	IQR	10.13	109,332
Daily E. coli	Warm-day count over prior 7 days	Site-specific P75	8.7%	1.4% to 16.5%	0.019	IQR	4.00	109,332
Daily E. coli	Warm-day count over prior 14 days	Site-specific P75	13.0%	4.5% to 22.2%	0.002	IQR	7.00	109,332
Daily E. coli	Warm-day count over prior 7 days	Site-specific P80	5.8%	0.0% to 11.9%	0.049	IQR	3.00	109,332
Daily E. coli	Warm-day count over prior 14 days	Site-specific P80	7.5%	1.5% to 13.9%	0.014	IQR	5.00	109,332
Daily E. coli	Warm-day count over prior 7 days	Site-specific P85	3.8%	-0.1% to 7.9%	0.058	IQR	2.00	109,332
Daily E. coli	Warm-day count over prior 14 days	Site-specific P85	5.3%	0.5% to 10.4%	0.032	IQR	4.00	109,332
Daily E. coli	Warm-day count over prior 7 days	Site-specific P90	1.9%	-0.0% to 3.8%	0.051	IQR	1.00	109,332
Daily E. coli	Warm-day count over prior 14 days	Site-specific P90	2.3%	-0.1% to 4.8%	0.063	IQR	2.00	109,332

Outcome	Metric	Threshold	Percent change over contrast	95% CI	P value	Contrast type	Exposure contrast	N observations
Daily turbidity	14-day mean temperature	Continuous	0.7%	-9.8% to 12.4%	0.905	IQR	13.07	203,566
Daily turbidity	Warm-day count over prior 7 days	Site-specific P75	-1.7%	-4.7% to 1.5%	0.301	IQR	4.00	203,566
Daily turbidity	Warm-day count over prior 14 days	Site-specific P75	-3.8%	-8.6% to 1.3%	0.140	IQR	7.00	203,566
Daily turbidity	Warm-day count over prior 7 days	Site-specific P80	-0.7%	-3.1% to 1.8%	0.593	IQR	3.00	203,566
Daily turbidity	Warm-day count over prior 14 days	Site-specific P80	-2.4%	-5.6% to 1.0%	0.168	IQR	5.00	203,566
Daily turbidity	Warm-day count over prior 7 days	Site-specific P85	-0.5%	-2.2% to 1.3%	0.592	IQR	2.00	203,566
Daily turbidity	Warm-day count over prior 14 days	Site-specific P85	-2.6%	-5.2% to 0.1%	0.064	IQR	4.00	203,566
Daily turbidity	Warm-day count over prior 7 days	Site-specific P90	-0.1%	-1.1% to 0.8%	0.767	IQR	1.00	203,566
Daily turbidity	Warm-day count over prior 14 days	Site-specific P90	-1.3%	-2.7% to 0.2%	0.085	IQR	2.00	203,566

Supplementary Table 18 | Daily HMS plume-arrival validation in transport-only smoke months. Daily *E. coli* models replace the county smoke proxy with direct NOAA HMS plume polygons linked to WQP site-days in transport-only smoke months. Same-day and lag-4 plume-arrival terms are shown separately for the 200–500 km and 500–1,000 km transport bands and for the pooled 200–1,000 km sample.

Outcome	Sample	Alignment	Percent change	95% CI	P value	N observations	N site-months	Counties
Daily E. coli	Goldilocks 200-1000 km	Same-day HMS plume	-14.1%	-21.3% to -6.2%	< 0.001	7,686	1,273	141
Daily E. coli	Goldilocks 200-1000 km	Lag-4 HMS plume	14.5%	6.1% to 23.5%	< 0.001	5,754	549	55
Daily E. coli	Goldilocks 200-1000 km	Same-day HMS density rank	-9.5%	-14.8% to -3.8%	0.002	7,732	1,296	141
Daily E. coli	Goldilocks 200-1000 km	Lag-4 HMS density rank	7.6%	2.5% to 12.9%	0.004	5,768	556	55
Daily E. coli	Transport band 200-500 km	Same-day HMS plume	-23.5%	-33.0% to -12.7%	< 0.001	3,303	576	92
Daily E. coli	Transport band 200-500 km	Lag-4 HMS plume	6.6%	-6.1% to 21.0%	0.333	2,377	253	39
Daily E. coli	Transport band 200-500 km	Same-day HMS density rank	-15.3%	-22.8% to -7.2%	< 0.001	3,325	587	93
Daily E. coli	Transport band 200-500 km	Lag-4 HMS density rank	2.5%	-6.0% to 11.8%	0.575	2,383	256	39
Daily E. coli	Transport band 500-1000 km	Same-day HMS plume	-6.8%	-15.6% to 2.9%	0.165	4,383	697	88
Daily E. coli	Transport band 500-1000 km	Lag-4 HMS plume	20.7%	11.5% to 30.6%	< 0.001	3,377	296	37

Outcome	Sample	Alignment	Percent change	95% CI	P value	N observations	N site-months	Counties
Daily E. coli	Transport band 500-1000 km	Same-day HMS density rank	-5.3%	-11.1% to 0.9%	0.096	4,407	709	88
Daily E. coli	Transport band 500-1000 km	Lag-4 HMS density rank	11.3%	5.1% to 17.8%	< 0.001	3,385	300	37

Supplementary Table 19 | Additional heat sharpening checks. Supplementary probes test whether same-day heatwave-day indicators or outage-vulnerability interactions sharpen the heat interpretation beyond the preferred rolling warm-day burden metric. Monthly subgroup slopes are shown for context because the main heat association is concentrated in groundwater systems and does not sharpen further under the more restrictive heatwave-day design.

Analysis	Sample	Specification	Effect metric	Effect	95% CI	P value	N observations
Daily WQP heatwave-day indicator	Daily E. coli	Site-specific P75, current >=3-day run	Percent change on heatwave day	4.9%	-2.3% to 12.6%	0.185	35,404
Daily WQP heatwave-day indicator	Daily E. coli	Site-specific P80, current >=3-day run	Percent change on heatwave day	4.9%	-2.0% to 12.4%	0.171	28,935
Daily WQP heatwave-day indicator	Daily turbidity	Site-specific P75, current >=3-day run	Percent change on heatwave day	-0.8%	-4.4% to 3.0%	0.674	65,072
Daily WQP heatwave-day indicator	Daily turbidity	Site-specific P80, current >=3-day run	Percent change on heatwave day	-1.1%	-4.8% to 2.8%	0.578	55,432
Monthly PWS heat slope by subgroup	System size	Large (>3,300)	RR per 1 C	1.048	1.018 to 1.080	0.002	116,415
Monthly PWS heat slope by subgroup	System size	Small (<=3,300)	RR per 1 C	1.023	1.004 to 1.042	0.016	554,391
Monthly PWS heat slope by subgroup	Ownership	Public	RR per 1 C	1.02	1.001 to 1.049	0.045	314,424

Analysis	Sample	Specification	Effect metric	Effect	95% CI	P value	N observations
				4			
Monthly PWS heat slope by subgroup	Ownership	Private	RR per 1 C	1.026	1.008 to 1.045	0.006	350,413
Monthly PWS heat slope by subgroup	Source type	Groundwater	RR per 1 C	1.029	1.009 to 1.049	0.004	566,327
Monthly PWS heat slope by subgroup	Source type	Surface	RR per 1 C	1.001	0.957 to 1.047	0.970	88,107
Monthly PWS heat x outage vulnerability	Low-vulnerability counties	Baseline slope	RR per 1 C	1.025		0.004	254,328
Monthly PWS heat x outage vulnerability	High-vulnerability counties	Baseline + interaction slope	RR per 1 C	1.011			254,328
Monthly PWS heat x outage vulnerability	Interaction term	High vs low outage vulnerability	Interaction RR per 1 C	0.986	0.978 to 0.995	0.001	254,328

Supplementary Figure 4 | Exploratory environmental justice gradients. a, Heat effect on violation risk stratified by county-level minority population quintile, shown as percentage change in violation risk by county-level minority population quintile. **b**, Burn scar × precipitation effect stratified by poverty quintile (three-way interaction *P* = 0.04), on the same percentage change scale. Panel b should be interpreted cautiously: the main burn scar effect is null (Extended Data Note 1), and a single significant three-way interaction among multiple tests is consistent with the expected false positive rate.

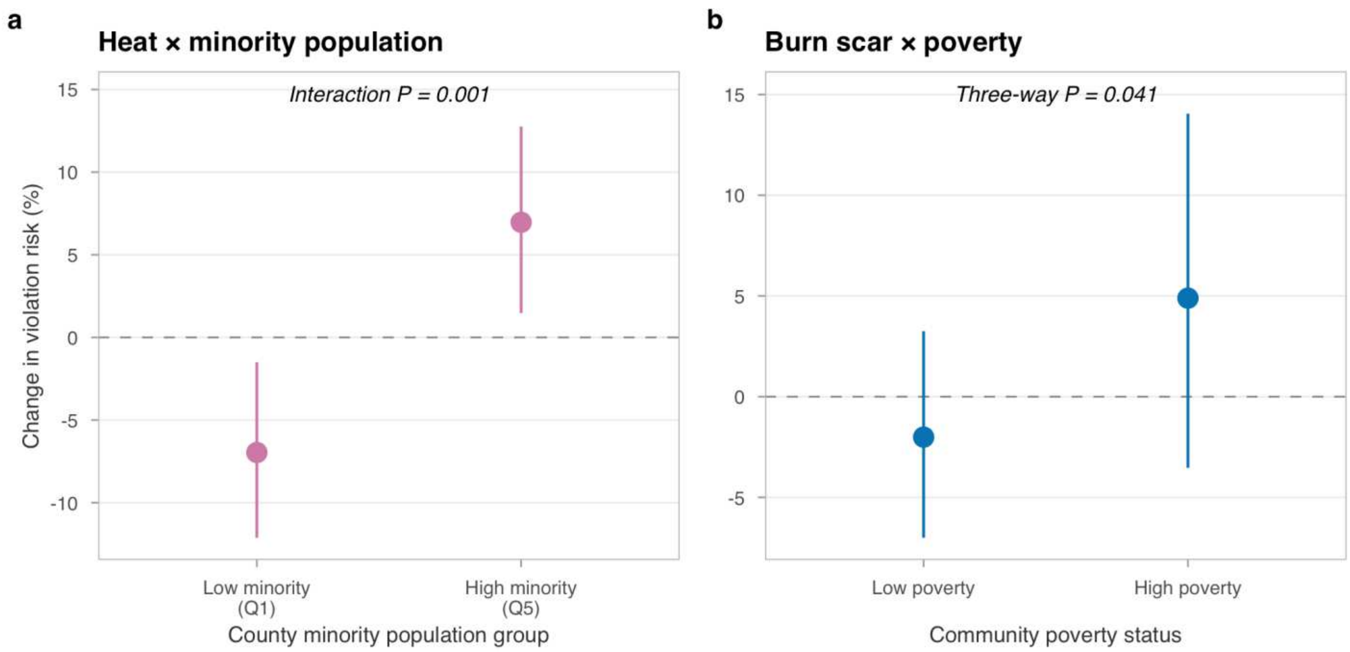

Supplementary Table 20 | Effective sample sizes by violation outcome. In fixed-effects Poisson estimation, systems with no within-panel outcome variation are separated from estimation. The estimand is therefore the treatment effect among ever-violating systems rather than a population-average effect. E. coli, the primary heat and smoke outcome, retains only 8.2% of systems (4,608 of 56,351). All models share the same total panel (56,351 systems, 9,021,476 system-months).

Outcome	Total systems	Ever-violating	Separated (%)	Effective system-months
Turbidity	56,351	26,012	53.8	4,388,794
Coliform	56,351	4,617	91.8	807,395
E. coli	56,351	4,608	91.8	805,775
DBP	56,351	14,074	75.0	2,422,764
Treatment technique	56,351	6,763	88.0	1,170,225
Health-based	56,351	21,708	61.5	3,714,187
Monitoring	56,351	39,938	29.1	6,721,926
SWTR	56,351	31,898	43.4	5,387,171
Nitrate	56,351	8,389	85.1	1,427,938
Arsenic	56,351	5,245	90.7	889,306
Lead/Copper	56,351	23,939	57.5	4,065,461
TOC	56,351	1,027	98.2	181,706
